# Patient and Healthcare Provider Barriers in the LDCT Lung Cancer Screening Continuum

**DOI:** 10.3390/diagnostics16071092

**Published:** 2026-04-04

**Authors:** Rodica Anghel, Antonia-Ruxandra Folea, Vlad-Luca Moga, Cristian Pavel, Diana Troncotă, Corneliu-Octavian Dumitru, Andreea-Iren Șerban, Liviu Bîlteanu

**Affiliations:** 1 Faculty of General Medicine, Carol Davila University of Medicine and Pharmacy, 8 Eroii Sanitari Street, 050474 Bucharest, Romania; rodica.anghel@umfcd.ro (R.A.); vlad-luca.moga@drd.umfcd.ro (V.-L.M.); cristian-pavel.pavel@drd.umfcd.ro (C.P.); diana.troncota@drd.umfcd.ro (D.T.); liviu.bilteanu@umfcd.ro (L.B.); 2 Oncological Institute “Alexandru Trestioreanu” Bucharest, 252 Soseaua Fundeni, 022328 Bucharest, Romania; 3 Remote Sensing Technology Institute, German Aerospace Center, Münchener Str. 20, 82234 Wessling, Germany; corneliu.dumitru@dlr.de; 4 Department of Preclinical Sciences, Faculty of Veterinary Medicine, University of Agronomic Sciences and Veterinary Medicine, 105 Splaiul Independentei, 050097 Bucharest, Romania; andreea-iren.serban@fmvb.usamv.ro; 5 Faculty of Biology, University of Bucharest, 91-95 Splaiul Independentei, 050095 Bucharest, Romania; 6 Laboratory for Molecular Nanotechnologies, National Institute for Research and Development in Microtechnologies—IMT Bucharest, 126A, Erou Iancu Nicolae Street, 077190 Voluntari, Romania

**Keywords:** lung cancer screening, low-dose computed tomography, uptake, longitudinal adherence, shared decision-making, smoking cessation, patient navigation

## Abstract

**Background/Objectives**: Despite demonstrated mortality benefits, annual low-dose computed tomography (LDCT) screening faces challenges in real-world adoption due to low uptake and poor longitudinal adherence. This review evaluates patient- and provider-level factors that influence screening participation and highlights strategies to strengthen equitable engagement throughout the screening pathway. **Methods**: A structured literature search of PubMed and Web of Science was performed to identify studies published between 2013 and November 2025 (search conducted on 25 November 2025). Eligible publications included qualitative and quantitative studies, study protocols, and reviews examining LDCT screening uptake, adherence, and follow-up practices. Extracted evidence was synthesized, with particular attention being paid to patient- and provider-level determinants. **Results**: The evidence demonstrates that both patient- and provider-level factors substantially influence screening participation and continuity. At the patient level, limited awareness of screening, misconceptions regarding asymptomatic disease, and psychosocial factors such as fear, fatalism, stigma, and medical mistrust were consistently associated with reduced uptake and adherence. At the provider level, gaps in guideline familiarity, time constraints, and challenges in delivering high-quality shared decision-making limited referrals and follow-up. **Conclusions**: Improving real-world effectiveness of LDCT lung cancer screening requires reframing screening as a longitudinal program of care. Strategies that support patient navigation, enhance provider capacity for sustained engagement, and integrate tobacco dependence treatment into screening pathways are central to improving adherence and reducing disparities.

## 1. Introduction

### 1.1. Background

Evidence from large-scale randomized controlled trials has demonstrated the efficacy of low-dose computed tomography (LDCT) in reducing lung cancer mortality [1,2,3,4] (see Table 1) in Western population [5,6] within the screening-eligible population [7,8,9,10,11,12] (see Supplementary Table S1). For example, it is established that annual LDCT screening significantly reduces lung cancer-specific mortality (by 20–24%) in high-risk populations compared to chest radiography (CXR) or no screening [13,14,15,16]. Across all trials [17,18,19,20,21], incidence in the eligible population is markedly higher than in the general population with a consistent order-of-magnitude increase, underscoring the strong concentration of lung cancer risk within screening-eligible groups and providing epidemiological justification for targeted lung cancer screening (LCS) strategies.

There is a clear inverse relationship where those with lower socioeconomic status (SES) and education levels have higher lung cancer risk but lower screening uptake [26,27,28]. Also, Black or African American individuals develop lung cancer at younger ages and with lower smoking intensities than White individuals, leading to disparities in eligibility under traditional guidelines [29,30,31].

The use of patient navigators and screening program coordinators significantly improves screening adherence and reduces time to treatment [28,32,33]. Mobile screening units successfully reach underserved, rural, and uninsured populations who otherwise would not access screening [27,34]. LCS is an established “teachable moment” for smoking cessation; screening combined with cessation interventions yields the highest mortality benefit [13,32,34].

### 1.2. Differentiation Between Uptake and Longitudinal Adherence

The distinction between initial uptake and longitudinal adherence is vital because real-world data demonstrates a sharp decline in participation after the baseline exam, particularly among vulnerable populations. For example, while a program might successfully recruit patients for a baseline scan, one study found that adherence to annual follow-up dropped to 23.7% at year one and further to 2.8% at year two among patients with negative baseline screens [35].

This phenomenon suggests that the factors driving initial uptake (e.g., provider recommendation, perceived risk) may differ from those sustaining long-term adherence (e.g., reminders, logistical barriers) [36]. Additionally, research indicates that inconsistent adherence is higher among patients with negative baseline scans compared to those with indeterminate results, suggesting a false sense of security or lack of understanding regarding the need for annual surveillance [37,38].

### 1.3. Objectives

This review aims to systematically analyze the factors that influence patient retention throughout the entire LCS framework. While the initial undergoing of an LDCT scan is the first critical step, the mortality benefit of LCS is predicated on repeated annual screening and the timely follow-up of detected nodules [13,37]. By categorizing determinants into patient and provider levels, this review will highlight where the “leaks” in the screening pipeline occur, from the initial order to the annual return, and identify specific interventions that sustain engagement [33,36].

## 2. Methodology: A Systematized Search Strategy

### 2.1. Search Strategy

**Figure 1:**
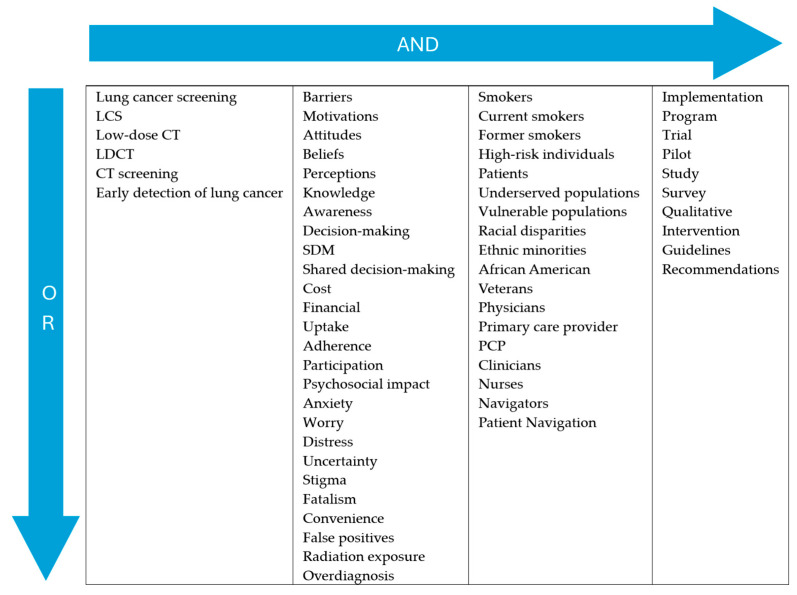
Search strategy detailing the keywords used and the order of Boolean operators (and/or) applied. The search formula was built upon terms like LCS or LDCT as broad topics, as shown in the first column, with the subsequent columns using more refined keywords: column 2 refers to challenges to screening, column 3 lists patient and provider-level stakeholders, and column 4 highlights initiatives. Boolean operators are applied column by column, from top to bottom within each column, proceeding sequentially from left to right. LCS—lung cancer screening; LDCT—low-dose computed tomography; PCP—primary care provider; SDM—shared decision-making.

This manuscript presents a narrative review; however, to ensure a comprehensive and transparent identification of relevant studies, the literature search was executed systematically using predefined search terms across multiple databases. The authors conducted a structured literature search in the PubMed and Web of Science databases, with studies published from 2013 onward considered for eligibility. The year 2013 was selected to reflect evidence emerging after the publication of the initial NLST results, as reported by the National Cancer Institute [39]. The search was carried out on 25 November 2025 by three reviewers, following the predefined search approach illustrated in Figure 1.

Within the search strategy’s stakeholder and population parameters (Figure 1, Column 3), broad terms (e.g., ‘Patients’, ‘Smokers’) were utilized to maximize overall search sensitivity. Simultaneously, specific demographic terms (e.g., ‘African American’, ‘Veterans’) were intentionally included to guarantee that targeted studies focusing strictly on highly vulnerable populations, who face unique and disproportionate screening disparities, were not inadvertently missed by the retrieval algorithm. The exact search formula (as per Figure 1) used on all databases with the required syntax adaptations is:

((“lung cancer screening” OR LCS OR LDCT OR “low-dose CT” OR “CT screening” OR “early detection of cancer”) AND (“barriers” OR “motivations” OR “attitudes” OR “beliefs” OR “perceptions” OR “knowledge” OR “awareness” OR “decision-making” OR SDM OR “shared decision-making” OR “cost” OR “financial” OR “uptake” OR “adherence” OR “participation” OR “psychosocial impact” OR “anxiety” OR “worry” OR “distress” OR “uncertainty” OR “stigma” OR “fatalism” OR “convenience” OR “false positives” OR “radiation exposure” OR “overdiagnosis”) AND (“smokers” OR “current smokers” OR “former smokers” OR “high-risk individuals” OR “patients” OR “physicians” OR PCP OR “primary care provider” OR “clinicians” OR “nurses” OR “navigators” OR “patient navigation” OR “underserved populations” OR “vulnerable populations” OR “racial disparities” OR “ethnic minorities” OR “African American” OR “Veterans” OR “VA healthcare system”) AND (“implementation” OR “program” OR “trial” OR “pilot” OR “study” OR “survey” OR “qualitative” OR “intervention” OR “guidelines” OR “recommendations” OR “policy”))

### 2.2. Inclusion and Exclusion Criteria

This review includes research articles, study protocols, and reviews that specifically examine uptake and longitudinal adherence to LDCT lung cancer screening, with an emphasis on patient- and provider-level barriers influencing participation. Included studies addressed patient-related factors affecting screening engagement (e.g., psychosocial responses, uncertainty regarding screening procedures, cancer-related worry) as well as healthcare professional-related factors (e.g., knowledge gaps, screening recommendations, and shared decision-making practices).

Studies focusing solely on CXR screening were excluded. To be considered relevant to the topic of LDCT screening adherence or uptake, studies must have explicitly measured, systematically reviewed, or qualitatively evaluated factors acting as specific barriers or facilitators to either the initiation of screening (uptake) or the continuation of annual surveillance (longitudinal adherence).

### 2.3. Data Extraction

Data extraction included quantitative measures of screening uptake, adherence, and follow-up, as well as qualitative findings describing patient and provider perspectives, experiences, and factors influencing engagement with LDCT lung cancer screening. Extracted data were synthesized to identify patterns related to patient- and provider-level barriers to uptake and longitudinal adherence.

### 2.4. Selection Process

**Figure 2:**
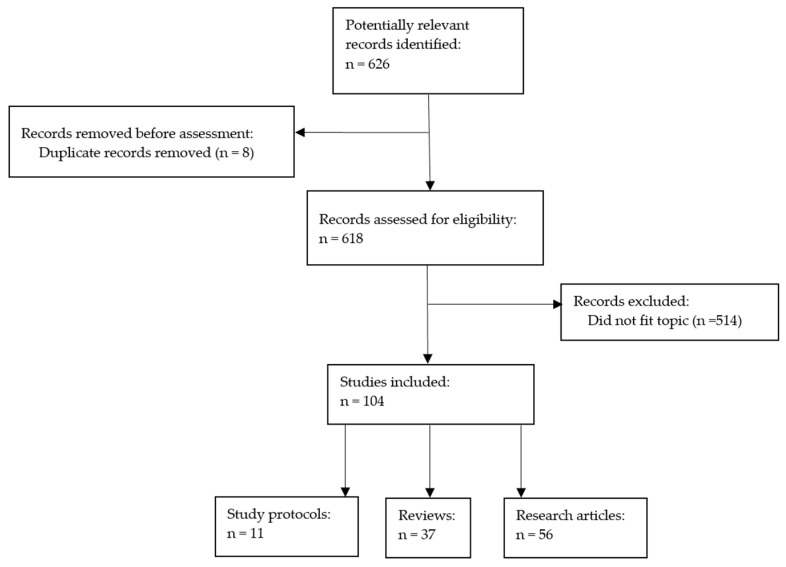
Flow diagram of study identification, assessment, and inclusion.

A schematic representation of the study identification and selection process is illustrated in Figure 2. Although this review is narrative in nature, the literature search was conducted using a structured and systematic approach, with predefined search terms applied consistently across databases to ensure a comprehensive and transparent identification of relevant studies.

Non-English studies, conference abstracts, letters or editorials were filtered out before any papers were screened.

As such, after the authors’ assessment of 626 identified records and the exclusion of subsequent ones that did not fit this review’s eligibility criteria, 104 studies were included in the final analysis, of which 37 were reviews, 56 were research articles and 11 were study protocols.

Study protocols (n = 11) were intentionally included in this review to showcase emerging and planned interventions specifically designed to address known barriers, providing insight into the future direction of LCS implementation.

## 3. Patient-Level Factors: Psychosocial and Socioeconomic Barriers

### 3.1. Knowledge and Awareness

#### 3.1.1. Lack of Awareness About Eligibility and the Concept of Screening

The lack of knowledge and awareness regarding LCS is a pervasive, multifaceted barrier to uptake involving both patients and providers. Research indicates that the “gap in the current state of the science in lung screening is population awareness and knowledge,” which contributes directly to low rates of patient–clinician discussions and screening uptake [40].

A fundamental impediment to adherence is the pervasive lack of awareness among high-risk individuals that LCS even exists or that they are eligible for it. Across multiple study populations, awareness of LCS remains “exceptionally low,” with recent estimates suggesting less than 30% of the screening-eligible population is aware that screening exists [41]. Unfamiliarity with LCS is common nationwide; studies have indicated that between 38% and 59% of smokers in the United States were unaware of the existence of screening programs [42], and this trend holds globally. For instance, a study in Karachi, Pakistan, found that only one-third of smokers had heard of LCS [43].

Detailed studies reveal the depth of this knowledge gap. In a study of current and former smokers, only 52% reported having ever heard of LCS, with awareness notably lower among current smokers compared to former smokers [44]. In a qualitative study of 105 smokers, only two participants were aware that CT scans could be used for screening; the vast majority were unaware such tests existed [45]. In a survey of smokers enrolled in a tobacco cessation program, nearly 84% of respondents had never heard of LCS, and no participant who had not already undergone the procedure was familiar with it [46]. Approximately 50% of these participants cited a “lack of knowledge about the test” as a barrier to screening [46].

This lack of awareness extends to a fundamental misunderstanding of the concept of screening versus diagnostic testing. Patients often do not know why LCS is being offered or what it entails [47]. Qualitative studies reveal that patients are often “bewildered” by the purpose of LCS and unable to distinguish it from diagnostic examinations [48]. Participants frequently described experiences with CXRs or diagnostic scans ordered for specific symptoms rather than preventative screening [45,48]. Even among patients who have already completed an LDCT scan, knowledge gaps persist; a study of Black Veterans found that most claimed to have little or no knowledge of the LCS process [49], and patients often did not realize that the CT scan they underwent was intended specifically as a screening test until explicitly told later [50].

Socioeconomic factors further exacerbate these gaps. Knowledge gaps are particularly prevalent among individuals with lower SES and lower health literacy [16]. Educational materials regarding LCS are often written at levels inaccessible to those with average health literacy [51], meaning individuals from marginalized groups are often less likely to be aware of screening benefits or their own eligibility [29].

Furthermore, the lack of awareness is not limited to patients; it also exists at the provider level. Providers often lack awareness regarding the availability of LCS and the specific eligibility criteria required to refer patients [33]. Contributing factors include a lack of clinician knowledge regarding screening guideline components and skepticism about the evidence base [52]. When primary care providers are unfamiliar with guidelines, they are less likely to initiate the necessary shared decision-making (SDM) conversations [42,53], leaving patients unaware that they qualify. Patients identify “not receiving a recommendation from a healthcare clinician” as a top barrier to participation [40].

#### 3.1.2. Misconceptions That “No Symptoms” Means “No Screening Needed”

A critical barrier to adherence is the conflation of health with the absence of symptoms. There are specific misconceptions among high-risk populations that LDCT screening is only needed when symptoms are present or if a CXR has not been performed [16]. This misconception fundamentally conflicts with the purpose of screening (detecting asymptomatic disease) and leads to delay or refusal.

The belief that one should only seek care when feeling ill is widespread. In a nationwide survey in France, 85% of the general population believed that symptoms are present in most cases of early-stage lung cancer [54]. In a survey of high-risk smokers, 40.2% of participants cited the “absence of symptoms of lung cancer” as a reason for not undergoing screening [46]. In a study of high-risk individuals in Puerto Rico, participants expressed difficulty accepting that one should participate in screening without symptoms, stating: “If I feel something strange in my body... well then [I ask to get screened]” [55].

Qualitative data reinforce this “risk denial” attitude; participants often believe that if they “don’t hear no rattle,” they are fine and do not require medical investigation [45]. This misunderstanding is sometimes reinforced by healthcare interactions where imaging is historically only ordered in response to a specific complaint, such as a bad cough [45]. In some cases, providers inadvertently reinforce this; for example, some PCPs in Puerto Rico believed that symptoms needed to be present before an LDCT test could be ordered [55].

This misconception also affects adherence to follow-up surveillance. Participants cited “feeling asymptomatic” as a reason for not returning for repeat annual screening, operating under the false belief that the absence of physical symptoms equated to an absence of risk [56]. Patients with indeterminate nodules often expressed confusion as to why a nodule was detected when they felt healthy [57].

Misconceptions are often rooted in fear rather than just a lack of data. Barriers include “fear of cancer diagnosis,” “cancer fatalism,” and “stigma” [47]. Targeted materials must provide a lay explanation for how early detection works to counter fatalistic beliefs [58] and address misinformation regarding affordability and medical mistrust [40,47]. To counter the “symptom fallacy,” programs must explicitly educate patients that they must be asymptomatic to qualify for screening, reframing the absence of symptoms as a prerequisite for screening rather than a reason to avoid it [13].

### 3.2. Psychosocial Beliefs and Stigma

#### 3.2.1. Fear and Fatalism

Psychological barriers significantly impede participation in LCS, with a primary defense mechanism being the “better not to know” phenomenon, also described as the “ostrich effect” or “knowledge avoidance” [59,60]. Research indicates that many eligible individuals prefer ignorance regarding a potential cancer diagnosis to avoid the associated psychological distress and anxiety [16]. In qualitative studies, participants have explicitly stated, “if I have lung cancer… basically I just don’t want to know about it” or expressed a “fear of the unknown” where knowing would trigger a “scary response” [59].

This sentiment was echoed in the UKLS trial, where “emotional barriers” were identified as a key reason for non-participation; individuals declining screening frequently endorsed the statement, “I do not wish to know if I had lung cancer, so I try not to think about it” [60]. This avoidance behavior is often characterized by a “risk denial” attitude, where focus group participants use phrases like “what I don’t know won’t hurt me” to rationalize their refusal [45]. Qualitative inquiries consistently reveal that barriers to screening are not merely logistical but are deeply rooted in psychological defense mechanisms and system distrust (see Table 2 for the classification of barriers and Supplementary Table S2 for patient quotes illustrating each barrier domain). This typology of “avoiders” often declines screening in safety-net settings because they simply “didn’t want to know” about lung cancer [61].

While fear can sometimes motivate health behaviors, in the context of lung cancer, it often acts as a paralyzing agent due to the high mortality and stigma associated with the disease. A deep-seated “fear of finding cancer” acts as a primary deterrent to seeking care [47,62]. This fear is pervasive: in a survey of current smokers, 52.5% cited “worrying about the results” as a reason to delay or avoid screening [46], while another study found that 33% of smokers refusing a CT scan explicitly cited being “afraid to find out whether they had cancer” [63]. Survey data corroborates the prevalence of these fears, quantifying the significant proportion of eligible individuals who delay screening due to anxiety and cost concerns (Table 3). The prevalence of worries about costs is about the same level (38.1% and 33.3%) according to two studies [46,63], while the same studies report different prevalences for fear of outcome (56.1% vs. 33.3%).

The fear of diagnosis is often linked to catastrophic thinking and the perception of lung cancer as a “death sentence” or “death knell,” rendering screening futile in the patient’s eyes [54,57,64]. In a study of high-risk individuals in Puerto Rico, participants expressed a belief that a diagnosis would lead to immediate death, stating, “they’ll tell me I have cancer and I’ll die,” or that one would “die within three days, just thinking of that” [55]. Furthermore, 47.4% of eligible smokers in one study indicated a belief that “treatment is more of a suffering than the disease itself” [46].

**Table 3:** Prevalence of perceived barriers to screening among high-risk screening-eligible populations [43,46,63,65,66].

Study Population	Barrier or Reason for Delaying or Avoiding Screening	Explanation		Prevalence (%)
Smokers aged 55–80 [46]	Current smoker	Perceiving current smoking status as a reason to delay		62.2%
	Lack of knowledge	About the test		56.4%
	Worry about result	Anxiety regarding potential findings		56.1%
	Lack of symptoms	Belief that lack of symptoms means screening is unnecessary		45.0%
	High cost	Concern regarding financial burden		38.1%
	Stigma/blame	Worry about being blamed for having smoked		36.9%
	Fear of harm from LDCT	Fear of radiation effects on health		9.0%
Current smokers declining screening [63]	Cost/no insurance	Cited as reason for not getting screened		33.3%
	Fear of finding cancer	Afraid to find out whether they had lung cancer		33.3%
Older smokers (aged ≥ 55) [66]	Fear of finding cancer	“Is afraid CT scan will find cancer”		51.3%
	Radiation fear	“Afraid radiation could cause lung cancer”		39.1%
	Fear of scanning process	“Scared of CT scans” (general fear of the procedure)		32.8%
	Fatalism	Belief that “The treatment is more of a suffering than the disease itself”		47.4%
Current/former Smokers (Pakistan) [43]	Health anxiety	“Screening would only make you feel more anxious about your health”		68.6%
	Financial cost	“Lung cancer screening is too expensive for you to afford”		61.4%
	Fear of positive result	“You’re afraid of a positive result”		52.6%
	Fear of hospitals/scanners	“Fear of hospitals and CT scanners prevent you from screening”		39.1%
LCS program participants (racial disparities) [65]	Cost	Percentage rating cost as a “Very Important” factor in decision-making	Black	58.4%
			White	37.8%
	Convenience	Percentage rating convenience as a “Very Important” factor in decision-making	Black	60.0%
			White	26.8%
	Risk of disease	Percentage rating risk of disease as a “Very Important” factor in decision-making	Black	61.4%
			White	45.1%

These fears severely impede a patient’s ability to process health information rationally. High levels of “affective risk perception” (worry and concern) are statistically associated with lower uptake of screening, suggesting that when the threat feels too overwhelming, it induces avoidance rather than proactive management [60,67]. Primary care patients identify “fear related to lung cancer” and “negative attitudes about screening” as significant obstacles, often resulting in risk aversion [52,58].

Fear is frequently compounded by fatalism, the worldview that intervention is futile because the health outcome is predetermined. Many high-risk individuals hold beliefs such as “the damage is already done” or that they have “smoked too long to benefit” from early detection [16,42]. This form of “medical nihilism” (the belief that treatment would be futile even if cancer were found) is particularly prevalent among marginalized groups and those with lower SES [29,31]. Fatalism can be reinforced by family history; for example, Black Veterans described avoiding screening because relatives had died shortly after diagnosis, reinforcing the belief that detection does not alter the outcome [49]. Additionally, the stigma associated with smoking reinforces these fatalistic views, as patients may feel shame or guilt, perceiving the disease as self-inflicted [16,34]. Contrary to the assumption that fear might motivate action, evidence suggests that high levels of distress and fatalism are associated with lower adherence to surveillance and follow-up protocols [68].

Anxiety extends to the screening process itself, known as “scanxiety.” Patients describe the fear of “going through the process of finding out” as a significant challenge [69,70]. Even among those who are screened, the fear of indeterminate results or a “false alarm” can generate significant psychosocial distress. The uncertainty associated with suspicious findings and subsequent follow-up procedures leads some patients to avoid the process entirely to escape this anxiety [69,71].

To address these barriers, targeted strategies have been designed to explicitly minimize fatalism and fear. Invitation materials may provide lay explanations of early treatment (e.g., illustrating that the lung is a treatable organ) to counter the view that screening is pointless [58]. Interventions like the “Lung Talk” tool are theoretically grounded to target variables like “fatalism” and “worry” [40], while the “Patient Voices” video was created to provide social support and normalize the choice to screen [62]. Additionally, baseline surveys for trials are increasingly including assessments of “cancer fatalism” and “medical mistrust” to better understand these barriers [47,52].

#### 3.2.2. Stigma

Lung cancer stigma refers to perceived health-related stigma characterized by exclusion, blame, or devaluation arising from anticipated negative judgment [72]. Unlike other malignancies often viewed as a “twist of fate”, such as breast or bowel cancer, where patients are typically viewed as blameless and deserving of empathy, lung cancer is socially represented and widely perceived as a “self-inflicted disease” or a “punishment” due to its strong behavioral link to smoking [16,34,54]. This perception generates a distinct psychological burden, with patients reporting higher levels of self-blame and guilt than those with breast or prostate cancer [72].

Stigma is not merely imposed from the outside; it is deeply internalized by smokers, manifesting as profound regret, guilt, and feelings of entrapment regarding their tobacco dependency [16,64]. Smokers describe themselves using strongly negative language, and internalized stigma is formally measured in cessation and screening research using validated tools such as the Internalized Stigma of Smoking Inventory [73,74].

Research indicates that feelings of guilt and shame are prevalent among the screening-eligible population. In a study of patients with lung cancer, over 50% agreed that the disease is viewed generally as self-inflicted (51.2%), and nearly 20% reported being explicitly told by others that their diagnosis was what they “deserved” for smoking [75]. Crucially, this stigma extends to those who have already quit; respondents reported that others assume lung cancer is caused by smoking even if the patient stopped smoking years ago or never smoked at all [75].

Stigma functions as a significant deterrent to seeking care and engaging in SDM. Patients report “experiences of smoking-related stigma” and the fear of being “labeled” or singled out as specific reasons for avoiding screening [47,58]. In a survey of current smokers eligible for screening, over one-third (36.9%) identified “worry about being blamed for having smoked” and “worry about feeling like a social outcast for smoking” (35.7%) as reasons for delaying screening [46].

Screening may be perceived as punitive rather than preventive [73], contributing to concealment of smoking history and potential diagnostic delay [16,34].

Furthermore, interactions with clinicians can exacerbate these feelings. Qualitative data reveal that smokers often feel treated unfairly by medical professionals [42,64]. If providers use alienating language or dismiss clinical findings (such as nodules) as merely the expected consequence of smoking, it can validate the patient’s fatalism and trigger disengagement from the healthcare system [50].

At a systemic level, stigma contributes to disparities in research funding and public support for screening initiatives [13,34]. Perceived lung cancer stigma is associated with poorer health outcomes, including higher levels of depression, greater symptom burden, and reduced health-related quality of life [75]. Visible symptoms, such as coughing, may further reinforce stigmatization through negative social reactions [75].

To mitigate these effects, interventions such as the “Patient Voices” video and stigma-sensitive invitation strategies frame screening as supportive and non-judgmental [58,62], sometimes intentionally avoiding explicit references to smoking to reduce feelings of accusation [58].

### 3.3. The Smoking “Teachable Moment”

#### 3.3.1. How Screening Impacts Motivation to Quit Smoking

LCS provides a unique and critical opportunity, frequently described in the literature as a “teachable moment”, to engage current smokers in cessation efforts [71,74]. This opportunity arises because the screening process creates recurrent interactions between high-risk patients and healthcare providers (up to six opportunities in one year), allowing for the assessment of smoking status and the delivery of counseling [13,22]. The screening event itself, from invitation to the receipt of results, serves as a cue that can heighten risk perception and prompt behavior change [76,77].

Research indicates that the process of undergoing screening generally enhances motivation and readiness to quit, regardless of the final result. In a study assessing smokers immediately before and after LCS, 25.7% reported increased readiness to quit shortly after receiving results, with overall motivation scores rising significantly [77]. Even before results were delivered, participants reported a significant decrease in the number of cigarettes smoked per day [77]. Empirical evidence supports the existence of a “teachable moment,” demonstrating immediate positive changes in cessation motivation following the screening event (Table 4).

This motivation is often driven by the emotional response to screening; “extreme worry” about lung cancer was associated with a higher likelihood of being ready to quit in the next 30 days [77]. Additionally, qualitative data suggest that smokers view LCS as a tool to “quantify and measure risks and harms,” expressing a desire to “see how much damage I’ve really done” to inform future decisions about quitting [73].

However, the relationship between perceived risk and motivation is complex. While “worry” increased readiness, high “perceived risk” was actually associated with being less likely to be ready to quit in some studies [77]. This paradox may be due to fatalism or low self-efficacy; smokers may believe they are at high risk but simultaneously hold a negative attitude toward their ability to quit.

Participation in LCS programs is generally associated with smoking cessation rates that are superior to those observed in the general population [13,16,34]. For instance, the NELSON trial reported a quit rate of 14.5% in the screening arm, and a Mayo Clinic study showed rates increasing from 14% to 24% over three annual examinations [13]. Despite this, actual engagement in formal cessation interventions can remain low. In one integrated program, only 25.5% of patients attended counseling appointments, with an actual quit rate of 11% [35]. Another study found that only 1.3% self-reported quitting in the short window between the scan and post-screening assessment [77].

#### 3.3.2. Mixed Evidence on Negative Scans: “License to Smoke” vs. Motivation to Quit

A critical debate in the literature concerns whether a “negative” (normal) screening result provides false reassurance (a phenomenon described as the “license to smoke” or “certificate of health”) or whether the screening process itself motivates cessation [13,16].

There is a theoretical concern that a clear scan may lead smokers to believe they are immune to the harms of tobacco or that screening “clears” them of damage. Qualitative studies suggest that some heavy smokers believe screening confers the same benefit as cessation [79] or that a negative result allows them to continue smoking [16]. In some studies, up to half of the evaluated patients reported decreased motivation to quit following screening, driven by such misperceptions [80]. Furthermore, trial attrition has been found to be higher among those with negative results [Lung Imaging Reporting and Data System (Lung-RADS) 1] compared to those with suspicious findings, suggesting these individuals may lose the urgency to engage in treatment [81]. Additionally, data from a Native American clinic found that those willing to engage in SDM had higher rates of ongoing commercial tobacco use (80%) than the sample at large (62%), suggesting complex interactions between engagement and behavior [82].

Conversely, the majority of evidence from large randomized trials (such as the NLST, NELSON, UKLS, and DLCST) suggests that the “license to smoke” phenomenon does not occur on a scale that negates screening benefits [13,34]. However, it is important to acknowledge that this evidence is primarily drawn from trial settings, and these participants may differ from the general population in their health consciousness, meaning these findings might not perfectly reflect real-world behaviors. Negative screening results were not associated with increased rates of smoking relapse among former smokers [13], and participants with negative results were still more likely to quit than those in unscreened control arms [24]. Longitudinal studies of NLST participants found that a negative test did not decrease risk perceptions or provide false reassurance [83]. Furthermore, changes in risk perception were not associated with changes in smoking status at 1-year follow-up [83], and specific screening results (negative vs. positive) were not statistically associated with changes in readiness to quit, suggesting the process of screening matters more than the result [77]. Survey data reinforce this, with one study finding that only 10.9% of respondents believed a negative result meant they could continue to smoke [16,46,66].

#### 3.3.3. Impact of Abnormal Findings and the Necessity of Integration

There is a direct correlation between positive or indeterminate screening results and increased rates of smoking cessation. Receiving an abnormal result makes the health risk salient, serving as a powerful motivator [13,34,80,84]. In the NLST, the odds of continued smoking were significantly lower for those with findings suspicious for lung cancer compared to those with negative exams [13]. Additionally, the identification of non-cancerous incidental findings on a “negative” cancer screen, such as emphysema or coronary artery calcifications, can serve as alternative “teachable moments” to motivate behavior change [76].

Ultimately, evidence suggests that undergoing screening alone “does not facilitate large-scale smoking cessation” without specific support [71,83]. While providers typically advise patients to quit, the majority fail to engage in the “supportive actions” necessary to drive change [85]. Consequently, guidelines emphasize that LCS must be paired with robust smoking cessation interventions to prevent false reassurance and maximize the “teachable moment” [67,74]. Integrating cessation with LDCT screening nearly doubles the reduction in lung cancer-specific mortality compared to screening alone and improves the cost-effectiveness of programs by 20% to 45% [13,22,34,35,84]. Patient navigation and motivational interviewing are recommended to harness this patient motivation and facilitate referrals to empirically supported programs [71].

### 3.4. Financial and Insurance Barriers

#### 3.4.1. Cost Concerns Persist Despite Coverage Expansion

Despite the expansion of insurance coverage for LCS following recommendations by the U.S. Preventive Services Task Force (USPSTF) and Centers for Medicare and Medicaid Services (CMS), financial and insurance issues remain a “formidable barrier” to uptake and adherence [13,42]. “Cost/insurance issues” are consistently identified as one of the top three perceived barriers to participation [40], and financial concerns remain a significant impediment even when coverage exists [52].

A significant disconnect exists between perceived and actual costs. Even among patients eligible for coverage without a co-pay, misunderstanding regarding financial liability is widespread. In qualitative interviews, patients frequently cited the expense of the test (estimating costs between $300 and $500) as a reason for opting out, unaware that their insurance would likely cover the procedure [45,59]. In a survey of smokers enrolled in cessation programs, 35.2% cited the “high cost of the study” as a barrier, despite most being insured and eligible [46]. Some patients misunderstood the “zero-dollar co-pay” provision or feared their insurance would not cover it, leading to avoidance [48]. Consequently, patient knowledge regarding the cost of screening is now a specific metric evaluated in trials [62].

#### 3.4.2. The “Diagnostic Gap” and Downstream Financial Toxicity

A critical financial barrier involves the costs associated with follow-up care rather than the initial screening scan. Educational materials often explicitly state that while the screening is covered, diagnostic testing for positive findings (such as nodules) will be billed to the patient’s insurance “in the customary manner,” meaning deductibles and co-pays apply [13]. Patients and providers express significant anxiety regarding these “downstream tests,” which may include additional imaging, biopsies, or bronchoscopy resulting from false-positive results or incidental findings [51,70,86].

This potential for out-of-pocket costs creates a fear of “financial toxicity” [70]. In focus groups, patients explicitly worried about the costs of “additional tests that would be necessary if the scan found something” [48]. This barrier is profound; some patients may undergo the initial screening but subsequently decline necessary therapy or follow-up due to economic barriers, thereby incurring the risks of screening (such as radiation and anxiety) without experiencing the survival benefits [29]. “Financial resources” are listed alongside logistical challenges as key reasons for delays in obtaining necessary diagnostic evaluation after an abnormal screen [87].

#### 3.4.3. Coverage Gaps and Variation

Systemic disparities in coverage further complicate access. While Medicare covers LCS for eligible beneficiaries, Medicaid coverage varies significantly by state. As of a 2021 review, only 31 of 50 state fee-for-service programs covered LCS, and the lack of Medicaid expansion in certain states exacerbates these gaps [26,29]. Furthermore, “grandfathered” health plans may still charge cost-sharing [88,89]. Within the Veterans Health Administration (VHA), while many receive screening at no cost, others are subject to a $50 co-pay depending on their benefit structure, which can influence willingness to participate [90].

For the uninsured or those seeking care at accredited imaging centers, the average out-of-pocket cost for LCS has been estimated at $583, with a range extending from $49 to $2409 [51,53]. In international contexts without universal coverage, cost is often the predominant barrier; for example, in Pakistan, the cost of an LDCT can exceed a monthly salary [43], and in Iran, it is the most frequently cited barrier [91].

#### 3.4.4. Indirect Costs, Administrative Barriers, and Mitigation Strategies

Financial barriers extend beyond direct medical billing to include indirect costs such as transportation, hospital parking fees, and lost wages from taking time off work [24,42,60]. Economic evaluations must account for “patient time and travel costs” [74], and “financial stressors” are recognized as barriers to smoking cessation, which is integral to the screening process [85].

Administrative hurdles also function as financial barriers. Providers report that insurance coverage denials and prior authorization requirements create logistical challenges [42,92]. For instance, providers in Puerto Rico noted that insurance companies sometimes deny LCS claims or require a CXR first, creating burdens for patients unable to afford out-of-pocket payments [55]. These factors correlate with lower adherence; patients with Medicaid or dual eligibility have higher odds of missing appointments compared to those with private insurance or Medicare [29].

To alleviate these concerns, providers are encouraged to practice cost transparency and inform patients about financial assistance programs [51]. Patient navigation programs are increasingly utilized to help patients address logistical barriers, including assistance with insurance co-payments and navigating prior authorizations [92]. Navigators often conduct systematic screenings for social barriers, such as the ability to pay for basic utilities or treatment, to connect patients with community services [47,87]. Targeted communication tools, such as “Lung Talk”, are also designed to explicitly address cost concerns and correct misinformation [40].

### 3.5. Screening Disparities in Black/African American Populations and the Role of Educational Attainment

#### 3.5.1. Adherence Among African American Populations and the “African American Smoking Paradox”

African American populations, particularly men, face a “disproportionate burden of lung cancer morbidity and mortality,” yet they experience notable disparities in screening utilization and adherence [52]. Research consistently demonstrates that African American patients experience lower rates of both initial screening uptake and longitudinal adherence compared to White patients [49,53,93].

Meta-analyses have indicated that White patients are significantly more likely to adhere to LCS recommendations than patients of other races, with one study reporting they were twice as likely to adhere [Odds Ratio (OR) 2.0] [94]. These meta-analyses reveal consistent demographic and behavioral predictors of non-adherence, highlighting significant disparities based on race, smoking status, and education (Table 5).

Another analysis confirmed that White participants had a significantly lower relative risk of nonadherence (RR 0.69) compared to non-White participants [56]. Even within safety-net health systems or equal-access systems like the VHA, Black patients had lower odds of being up-to-date with screening and completing the initial process compared to White patients [31,97].

Self-reported Black race has been associated with 1.5 times the odds of missing LCS appointments compared to self-reported White race, even when controlling for other social determinants of health (SDOH) [29]. Furthermore, Black patients are more likely to be lost to follow-up after an initial baseline screen or a positive finding. For example, in a study of patients with Lung-RADS 3 findings (requiring short-term follow-up), a significant portion of Black patients did not present for the required imaging [28], and Black race has been associated with a significantly lower likelihood of follow-up after a positive LDCT screen independent of other factors [53].

A fundamental barrier to uptake is the “African American smoking paradox.” Research acknowledges that Black smokers often have lower smoking intensity (fewer cigarettes per day) and intermittent patterns compared to White smokers, yet they experience greater morbidity and mortality [52]. Consequently, standard eligibility guidelines (such as the USPSTF or those based on the NLST) often exclude a higher proportion of high-risk Black individuals because they do not meet heavy pack-year thresholds despite their elevated risk [30,52,93]. This creates a mismatch where Black smokers develop lung cancer at younger ages and with lower cumulative exposure than the criteria require [26]. Additionally, the high prevalence of menthol cigarette use, driven by race-based marketing, contributes to lower quitting success and increased cancer rates [52].

#### 3.5.2. Disparities Among Those with Lower Educational Attainment and SES

SES, often linked to educational attainment, is a strong predictor of screening participation. This reflects the “inverse care law,” where lung cancer risk is highest within lower SES communities, yet these populations show the lowest participation in screening programs [67].

Lower educational attainment is consistently associated with lower participation and adherence to LCS [60]. Completion of 4 or more years of college is associated with increased adherence (OR 1.5) [94], while a lack of post-secondary education predicts nonadherence [56]. In one study, only 37.5% of those with the lowest education level participated annually compared to 60.8% of those with higher education [78]. Furthermore, attrition in smoking cessation trials within the LCS setting was significantly higher among those with a high school education or less [81].

These disparities are exacerbated by health literacy gaps. Individuals with lower education levels have been found to have lower knowledge scores regarding lung cancer and screening [26], and report greater uncertainty regarding the reason for their referral [98]. Educational materials are often written at levels too complex for those with average or low health literacy [30], leading to confusion and risk aversion [58]. Consequently, illiterate patients have been found to be significantly less interested in participating in screening compared to those with college education [91].

There is also a concern regarding representation bias; major trials like the NLST had participants with higher education and SES than the general U.S. population, suggesting that the high adherence rates observed in trials (95%) may not be representative of real-world, medically underserved populations [58,71].

There is evidence of a significant interaction between race and education. One study found that African American participants with education beyond high school had significantly higher odds of adherence (OR 2.55) compared to White participants with lower education. This suggests that higher educational attainment may serve as a protective factor facilitating adherence specifically within the African American population [65].

#### 3.5.3. Race and Screening Program Structure

Programs utilizing dedicated navigators (centralized models) eliminate racial disparities compared to those relying solely on primary care providers (decentralized models). While Black race was associated with a 27% reduction in adherence to annual LCS at decentralized programs, this racial disparity was eliminated in centralized programs, where no significant difference in adherence was observed [38].

Barriers contributing to these disparities include medical mistrust, lack of physician referrals, and fears associated with cancer diagnosis [47,52]. Additionally, African American participants were significantly more likely than White participants to cite “convenience” and “cost” as very important factors in their decision to screen, and those prioritizing these factors had lower odds of adherence [65].

## 4. Provider-Level Factors: The Gatekeepers

### 4.1. Knowledge and Guideline Familiarity

#### 4.1.1. Gaps in Primary Care Provider (PCP) Knowledge Regarding Eligibility Criteria Leading to Under-Referral

PCPs serve as critical gatekeepers for LCS, yet a significant barrier to implementation is their lack of familiarity with specific screening guidelines and eligibility criteria. Research indicates that clinician knowledge gaps, specifically a “lack of knowledge about screening guideline components,” directly contribute to the low utilization of LDCT screening [52,92]. Many PCPs struggle to recall the exact inclusion criteria derived from major trials; in qualitative studies, providers were often unable to immediately recall specific age limits or pack-year thresholds, instead relying heavily on electronic health record (EHR) alerts to identify patients [99]. While primary care providers are tasked with the bulk of screening referrals, significant disparities exist between their confidence and knowledge regarding screening guidelines compared to their specialist counterparts (Table 6).

This lack of familiarity manifests as a significant confidence gap between generalists and specialists. A survey comparing providers found that PCPs were significantly less likely than specialists (pulmonologists, oncologists, radiologists) to feel confident in their ability to identify appropriate patients for screening (63.8% vs. 93.5%) [100]. In a survey of Los Angeles County PCPs, only 47% were even aware that LDCT was recommended by the USPSTF [42].

These knowledge gaps often lead to discordance between clinical practice and guidelines, resulting in both under-referral and inappropriate referrals. Confusion regarding the “multiplicity of guidelines” contributes to this issue, as PCPs cite difficulty keeping up with varying criteria alongside other preventative care requirements (“guideline fatigue”) [13,99]. Consequently, referrals often fail to meet established criteria. An analysis of referrals in Seattle found that 24% of referred patients were actually ineligible, often due to not meeting age requirements [42]. Furthermore, PCPs report “inadequate knowledge of the trade-offs of LCS,” which hampers their ability to conduct the necessary SDM [102].

#### 4.1.2. Fundamental Misunderstandings of the Screening Concept

The lack of awareness extends to the fundamental purpose of screening. A study of French physicians found that 93% of GPs used inappropriate tests (such as CXRs) for screening. Unlike specialists who targeted high-risk heavy smokers, 55% of GPs proposed screening for all smokers regardless of pack-year history, demonstrating a lack of familiarity with risk-stratification [101].

#### 4.1.3. Confusion Regarding Management of Incidental Findings

The management of screening results, including pulmonary nodules, false positives, and incidental findings, represents a significant source of anxiety and confusion for PCPs, often acting as a barrier to the initiation of screening programs [13]. PCPs describe the volume of abnormal results for which they are responsible as “staggering” [87]. In one analysis, 60% of screened patients had a false-positive result and 56% required tracking for nodules, leading providers to express concern about the anticipated burden of managing this workflow [42].

PCPs often feel ill-equipped to handle these findings. Compared to specialists, they report significantly lower confidence in deciding the appropriate work-up for positive CT findings (52.9% vs. 93.5%) [100]. Surveys reveal that 68% of providers needed additional information on follow-up recommendations for nodules, and 50% were “unsure” or “didn’t know” if Lung-RADS classifications were important for follow-up [42]. Clinicians have explicitly expressed a “need for more support” in managing these results [47].

#### 4.1.4. Complexity of Incidental and Non-Target Findings

Unlike mammography, LCS frequently detects “non-target” incidental findings, such as coronary artery calcifications, emphysema, fibrosis, or extrapulmonary malignancies, which adds complexity and cost to the evaluation [13,67,76]. In a study on direct access to LDCT, 30.9% of scans raised suspicion of other lung diseases and 18.4% raised suspicion of non-lung diseases, creating a high volume of incidental information for GPs to manage [103].

This complexity creates “uncertainty about responsibility” regarding who should manage the follow-up, the PCP or a specialist [87]. Most practices lack integrated tracking systems to ensure patients do not fall through the cracks [87]. Due to time constraints and low confidence, PCPs often prefer to “offload” this workload to screening coordinators or pulmonologists [99], and they emphasize the need for standardized language in radiology reports to guide next steps [70].

The consequences of this uncertainty are significant. PCPs admitted in interviews that they seldom provided detailed information about risk or surveillance plans to patients with incidental nodules, sometimes avoiding the topic to prevent distress [68]. While correct management can increase benefits, incorrect management of incidental findings risks over-investigation and harm to the patient [23].

### 4.2. Time Constraints in SDM

#### 4.2.1. Universal Time Pressures in Primary Care Practice

Time constraints represent a fundamental and widely documented limitation of shared decision-making (SDM) in primary care, extending beyond any specific healthcare system. In routine clinical practice, consultations are typically limited to 15–20 min, during which physicians must address multiple competing priorities [61]. This structural limitation is particularly pronounced in the population eligible for lung cancer screening (LCS), which is characterized by advanced age, heavy smoking history, and a high burden of comorbidities [92].

Clinicians consistently report that “there is always something else that’s taking priority,” resulting in preventive interventions such as screening being reduced to brief, often superficial exchanges or omitted entirely [88,99,104]. In this context, SDM competes with acute complaints, chronic disease management, and administrative tasks, creating a persistent tension between ideal and feasible care delivery [47,104].

These constraints have direct consequences on the quality of decision-making. Observational studies indicate that screening discussions in real-world settings may last as little as 57 s, with limited discussion of potential harms and minimal use of decision aids (DAs) [105]. To cope with time limitations, clinicians may prioritize the communication of benefits while omitting complex concepts such as overdiagnosis or false positives, which are perceived as time-consuming or potentially overwhelming for patients [104]. Furthermore, insufficient time for continuous education contributes to knowledge gaps regarding current recommendations, which may delay or hinder referral decisions [13].

Economic and organizational factors further reinforce these pressures. Physicians may intentionally shorten consultations due to productivity constraints, thereby limiting opportunities for in-depth discussion and patient engagement [13]. These dynamics collectively reflect structural characteristics of modern primary care systems and are not specific to a single national model.

#### 4.2.2. U.S.-Specific Regulatory Constraints: The CMS SDM Requirement

Superimposed on these universal time pressures, the U.S. healthcare system introduces an additional layer of complexity through the Centers for Medicare & Medicaid Services (CMS) mandate for LCS reimbursement. CMS requires that coverage be contingent upon a formally documented SDM visit, which must include eligibility assessment, use of a patient decision aid, smoking cessation counseling, and a balanced discussion of benefits and harms (including false positives, overdiagnosis, and radiation exposure) [35,75,85,87].

While conceptually aligned with high-quality patient-centered care, this requirement creates a significant operational burden within the constrained time framework of primary care. Providers report that fulfilling all mandated components is “not feasible” within standard consultations [61]. Even though clinicians estimate that a comprehensive screening discussion requires approximately 5–10 min, this time is rarely available in practice [52,61]. Survey data further highlight this discrepancy: only 14.3% of primary care physicians (PCPs) report having sufficient time for counseling, compared to 50% of specialists [100].

As a result, the regulatory requirement may inadvertently promote a “check-box” approach to SDM, where documentation is prioritized over meaningful deliberation [61]. Stakeholders also note a misalignment between reimbursement and actual resource utilization, with compensation (approximately 70) falling short of the estimated cost of delivering a complete SDM visit (approximately 130), potentially discouraging full implementation [42].

#### 4.2.3. Structural and Organizational Modifiers of Time Constraints

The impact of both universal and regulatory time pressures varies according to care delivery models. In decentralized systems, where PCPs are responsible for conducting SDM within routine visits, the time burden is particularly acute [61,105]. In contrast, centralized screening programs that employ dedicated personnel or structured workflows may allocate sufficient time for SDM, thereby improving the depth and quality of discussions [61,105].

To address these challenges, several alternative care models have been proposed. These include redistribution of professional roles, with trained non-physician staff conducting SDM, as well as the use of telemedicine to perform SDM encounters prior to in-person consultations [47,105]. Such approaches aim to reconcile the ethical and clinical imperatives of SDM with the operational realities of contemporary healthcare systems

### 4.3. Quality of Communication

#### 4.3.1. Clinician Overemphasis on Benefits vs. Harms

Research indicates a significant imbalance in how clinicians present LCS to patients, often prioritizing the potential for life-saving detection while minimizing associated risks. While guidelines require a balanced discussion, research indicates that SDM conversations regarding LCS often fail to meet minimum quality criteria, specifically regarding the balanced presentation of information [47].

Clinicians have reported focusing primarily on the benefits of screening, specifically reduced lung cancer mortality, with little acknowledgment of potential harms [104,105]. Analysis of patient-reported conversations reveals that benefits are discussed far more frequently than harms [105]. Providers often “overemphasize the benefits of LCS with little mention of the harms,” such as anxiety from findings, complications from downstream procedures, overdiagnosis, false positives, and radiation exposure [33,47,92].

Some clinicians explicitly stated that because they personally felt the benefits outweighed the risks, they did not “go into that so much with the patients” [104]. Others felt that discussing complex concepts like overdiagnosis or false positives was “way too much information” and could cause patients to disengage or feel overwhelmed [61,104]. Additionally, some clinicians actively avoid using the word “cancer” to prevent patient distress; however, qualitative studies show that patients generally want this information and find it reassuring rather than anxiety-provoking when risks are explained clearly [68].

Providers report “inadequate knowledge of the trade-offs of LCS,” which limits their capacity to effectively weigh benefits against risks with the patient [102].

Consequently, patients often perceive that there are no risks involved. In qualitative interviews, patients reported being told that there was “no downside” to the test, believing the benefits are “astronomical” while the risks are negligible or nonexistent—a “win-win situation” [73,104]. In focus groups, patients reported that their doctors were “very vague” about the screening tests, leaving them unaware of limitations [45].

Because meaningful dialogue is often missing, patients are frequently unprepared for abnormal results or the rationale for active surveillance (waiting and watching) rather than immediate biopsy. This lack of understanding contributes to significant distress, frustration, and confusion when indeterminate nodules are detected [58,68,104]. In contrast to routine practice, high-quality clinical trial protocols (e.g., the Yorkshire Lung Screening Trial) explicitly structure the SDM process to include a discussion of specific harms alongside benefits [67].

#### 4.3.2. Reliance on “Check-Box” SDM Rather than Meaningful Dialogue

While CMS mandates an SDM visit for reimbursement, an unprecedented requirement for preventive services, the implementation often resembles a bureaucratic hurdle rather than a substantive clinical interaction [102].

The current implementation of SDM often prioritizes information exchange (meeting CMS billing requirements) over the elicitation of patient values and preferences [61]. Some clinicians admitted they only used decision aids because of the CMS requirement, stating, “if I wasn’t required to, I probably would not be using any... tool” [104].

SDM in clinical practice is often “cursory” [92]. As noted previously, these interactions are often brief; however, the primary deficit is the focus on information exchange (‘option talk’) rather than exploring patient values (‘decision talk’) [105].

True SDM involves exploring patient values (“decision talk”), yet interactions are often information-centered (“option talk”) or deferred entirely to the provider’s recommendation [61]. Qualitative analyses reveal that patients frequently do not recognize that a decision has even been made, often ceding authority with sentiments such as “Whatever you say, Doc,” or feeling pressured to accept screening [68,73,104]. This lack of meaningful dialogue is evidenced by the fact that many veterans did not recall having an SDM discussion despite documentation of these events in their EHR [49].

Time constraints in primary care are a major driver of this superficial communication. PCPs report “insufficient time to engage in SDM” and “limited time per patient” (standard visits often filled with competing priorities) as major barriers to conducting adequate counseling [52,102,104]. Additionally, providers perceive barriers such as difficulty accessing DAs and limited patient comprehension [42]. Consequently, some experts argue that the CMS requirement for a dedicated SDM visit acts as a “barrier to screening” rather than a facilitator [47].

### 4.4. Therapeutic Alliance

#### 4.4.1. The Role of Trust in the Provider as a Primary Motivator for Screening Completion

The therapeutic alliance, specifically the trust established between the patient and the healthcare provider, is identified as a critical lever and a “decisive role” in the uptake and completion of LCS [106]. For many patients, particularly those who are unaware of LCS prior to a clinical visit, the decision to screen is driven primarily by their reliance on the provider’s judgment and the quality of their established relationship, rather than a deep understanding of medical statistics or the procedure itself [107].

Qualitative studies consistently identify “trust in the referring clinician” as a primary theme motivating adherence [107]. Patients describe an “eagerness to follow provider recommendations” based on mutual trust [49], noting they would undergo screening simply because “my doctor recommended it” or encouraged them to “do the right thing” [55,107]. This trust often acts as a substitute for medical knowledge; patients frequently do not understand specific risks or surveillance plans but rely on the belief that a “good doctor” would notify them if the situation was serious [68].

#### 4.4.2. Deference to Provider Judgment for Mitigating Distress and Ensuring Adherence

This reliance often manifests as patients “ceding decision-making” to their providers, respectfully yielding to the clinician’s expertise regarding complex trade-offs [73]. One participant noted, “I did what he said. I know he is badgering me for my own good” [71]. Clinicians report that many patients, particularly older adults, explicitly request a firm recommendation rather than engaging in detailed deliberation, viewing this guidance as a fundamental component of the doctor–patient relationship [48,61]. Consequently, physician recommendation remains the strongest predictor of screening utilization [34,100], and patients view one-on-one discussion with their physician as the most trusted mode of learning about LCS [43].

A strong therapeutic alliance functions as a coping mechanism that mitigates the psychological burden of screening [108]. High levels of trust explain why some patients remain satisfied with their care and experience less distress despite the uncertainty of pulmonary nodules [68]. Communication styles that emphasize the “patient as a person”, showing interest in the participant’s life and fostering partnership, are statistically associated with decreased patient distress and increased adherence to surveillance recommendations [68,108]. Conversely, a failure to ensure appropriate diagnostic testing following an abnormal result is described as a system failure that “violates the trust that patients place in their providers” [87].

#### 4.4.3. Addressing Mistrust and Disparities

While trust facilitates uptake, “medical mistrust” and “mistrust of the health care system” are significant barriers that deter engagement, particularly among racially heterogeneous and marginalized populations [47,52]. Black and Hispanic populations report higher levels of mistrust due to generational histories of discrimination and medical exploitation [29,64].

However, specific trust in a provider can mitigate broader systemic mistrust; patients may harbor “strong trust” in their specific clinic even if they distrust the system at large [82]. There are several types of interventions designed to leverage this. Trials like mFOCUS include specific “phone calls to build trust” alongside logistical support [87].

The SHARED project utilizes “citizen scientists,” and other programs employ community health workers (CHWs) or patient navigators who share the racial or social background of the target population to enhance relatability and trust [52,92,109].

Leveraging existing trusted relationships in primary care is hypothesized to be more effective for groups like Indigenous Māori than centralized screening models, which may prohibit the development of trust typically built through past encounters [106,109].

While qualitative data strongly supports trust as a motivator, quantitative associations can vary. One study found that while trust was a key qualitative theme, it did not significantly predict screening completion in a quantitative analysis of the same health system, suggesting the relationship may be complex or mediated by other factors in insured populations [41]. Nonetheless, the consensus remains that the therapeutic alliance is a decisive factor in engaging high-risk individuals.

## 5. Facilitators and Interventions: Strategies to Improve Adherence

### 5.1. Patient Navigation

#### 5.1.1. Reducing Barriers and Bridging Gaps in SDOH

Patient navigation serves as a proactive, “barrier-focused” intervention designed to guide patients through the complex LCS continuum, from initial screening to diagnosis and treatment [28,71,88]. Unlike standard clinical staff, navigators provide “instrumental (task-oriented or logistic) support” to identify and eliminate individual impediments to care [71].

Navigators actively assist with practical obstacles such as appointment scheduling, providing reminders, and coordinating follow-up care [88,110]. In a study of community health centers, navigators were responsible for scheduling screening CTs for 64% of the intervention group and provided reminders to 47% [110]. They also resolve transportation issues (e.g., arranging ride-sharing or van services) and help with expenses like parking [42,88].

Navigators help patients navigate the complexities of insurance coverage and cost concerns, which are major deterrents for underserved populations [28,88]. In the TELESCOPE trial, non-clinical navigators are tasked specifically with addressing “cost concerns” and answering “questions about insurance” [47]. In safety-net systems, navigators successfully addressed insurance issues for 29% of patients to resolve coverage gaps [88].

Beyond logistics, navigators address psychosocial barriers by offering emotional support to manage “cancer-related distress,” anxiety, and depression associated with screening procedures or anticipated results [28,88]. They employ motivational interviewing to empower patients and address fatalism or fear [110]. Furthermore, navigators may call patients shortly after result letters are sent to ensure comprehension, acting as a safety net for patient understanding [13].

Navigators are uniquely positioned to conduct systematic screenings for social barriers such as housing instability, food insecurity, and utility costs that disproportionately affect vulnerable populations [87]. Systematic screening for social determinants is essential for resolving these physical obstacles [87]. This also includes arranging subsidized phone services for patients who might otherwise be lost to follow-up [33].

#### 5.1.2. Impact on Uptake, Adherence, and Equity

Navigation has been proven to improve screening rates and eliminate disparities. Randomized controlled trials have demonstrated that navigation significantly increases screening uptake among current smokers (23.5% with navigation vs. 8.6% usual care) [33,110]. In safety-net systems, completion rates were higher in centralized programs utilizing navigators (63%) compared to decentralized settings that do not utilize dedicated staff to track and guide the screening process (56%) [96].

Structural support provided by navigators can eliminate racial disparities in adherence. Patient navigators successfully bridge gaps in care by managing orders and reminders, a strategy proven to neutralize the adherence disparities typically observed between Black and White patient populations [38]. In safety-net trials, navigation was particularly effective in addressing communication gaps early in the process; provider-related barriers reported by patients decreased by 85% over the course of the intervention [88].

#### 5.1.3. Efficacy in Vulnerable Populations

In the INHALE trial, navigation significantly increased LCS completion rates compared to usual care for those currently experiencing homelessness (26.8% vs. 7.1%) and those formerly homeless (51.3% vs. 10.2%) [111]. However, the effect was smaller for current homeless populations due to the “digital divide” (lack of cell phones) and competing subsistence priorities (food, shelter) [111].

Navigators mitigate barriers related to low health literacy and facilitate access to mobile units in rural areas [53,93].

Culturally competent navigators who share the racial, ethnic, or social background of the target population help build trust and mitigate medical mistrust [33,92]. Bilingual navigators effectively bridge communication gaps; in one study, Spanish-speaking patients navigated by bilingual staff reported fewer system-level barriers, though calls were approximately 30% longer due to the intensive support required [88].

#### 5.1.4. Distinction from System-Level Coordinators

Screening coordinators (often nurses) focus on clinical processes, such as managing registries, verifying eligibility, ordering scans, and tracking results for reimbursement [13,33]. In contrast, patient navigators (often laypeople or CHWs) are patient-facing and focus on “empowering the patient” to access that care through problem-solving and education [70,71].

Navigation is distinct from automated population health management (e.g., portal messages) because it adds personal, “high-touch” phone calls designed to build trust [87]. Some models distinguish between patient navigators (non-clinical staff addressing access/cost) and nurse navigators (clinical staff managing orders and abnormal findings) [47]. Navigators relieve the burden on time-constrained PCPs by assessing eligibility, introducing SDM, and updating the EHR [38,110].

### 5.2. DA Effectiveness

DAs are evidence-based tools designed to facilitate SDM by providing information about options, benefits, and harms, thereby helping patients clarify their values. Recent research highlights a transition from standard print materials to interactive, multimedia formats.

#### 5.2.1. Video and Web-Based DAs

Video- and web-based decision aids (e.g., “Lung Talk”, “ShouldIscreen.com”, “Patient Voices”) have been shown to improve patient knowledge, decisional quality, and preparedness compared with standard informational materials [23,45,95]. However, evidence regarding their impact on actual screening uptake remains mixed, with emerging data suggesting that tailoring these tools to smoking status may help address specific concerns and fears [112,113].

“Lung Talk” is a computer-tailored health communication and decision support tool designed to function as a “cue to action.” It is an interactive program that takes 8 to 12 min to complete, utilizing embedded audio, video, and animation segments [40,105,113].

“Patient Voices” is a 3 min and 23 s video intervention accessible via a private website. It features patient testimonials designed to manifest empathy and normalize the screening process for adults who may experience tobacco-related stigma [62].

Developed by the University of Michigan, “ShouldIscreen.com” is a web-based tool that includes a personalized lung cancer risk calculator (like PLCOm2012), recommendations, graphics, and sample scripts to support SDM discussions at the point of care [102,105,114]. “ScreenLC.com” is a personalized, web-based decision support tool used to provide risk estimates and support SDM discussions [102]. Video DA “Lung Cancer Screening: Is It Right for Me?” is a 9.5 min video DA tested in large randomized trials [115].

#### 5.2.2. Increasing Knowledge, Decisional Quality, and Preparedness

Research consistently demonstrates that multimedia and tailored aids are superior to standard educational materials in preparing patients for SDM. Users of DAs consistently show greater knowledge of LCS eligibility, benefits, and harms (e.g., false positives, overdiagnosis, radiation) compared to controls [105,112,115]. For instance, the “LCSDecTool” resulted in a sustained increase in knowledge immediately post-intervention that persisted at 1 and 3 months [112]. Similarly, participants who viewed “Lung Talk” demonstrated significantly greater improvements in knowledge compared to those who received a non-tailored information sheet [113].

DAs significantly reduce decisional conflict (uncertainty about the course of action) and increase patients’ clarity regarding their personal values [47,115]. Video-based aids specifically have been shown to help patients feel “well prepared” to make a screening decision (67.4% vs. 48.2% for standard brochures) [105,115]. Comparative Nuance: While effective, the format matters. In a trial comparing “ShouldIscreen.com” to a paper-based “Option Grid,” both tools resulted in high patient-reported SDM scores. However, participants using the web-based tool demonstrated significantly less knowledge regarding the potential complications of follow-up testing (63.4% correct vs. 84.8%) and reported higher decision regret compared to the paper-based group [114]. Some vulnerable populations preferred paper materials due to discomfort with computers [48].

#### 5.2.3. Mixed Evidence on Screening Uptake

While DAs consistently improve the quality of the decision-making process, their impact on the actual rate of screening uptake is mixed. Some trials show significant increases in uptake. A trial of the “LCSDecTool” integrated into primary care visits found that LCS uptake was significantly higher in the intervention group compared to the control group (37.7% vs. 21.1%) at 6 months, suggesting efficacy when coupled with a clinical encounter [112]. Preliminary data for “Lung Talk” also suggested a potential increase in uptake (31% vs. 10%) [40].

Conversely, several studies found no significant difference in screening completion rates. The trial of the “Lung Cancer Screening: Is It Right for Me?” video found no difference in scheduling or completion between the video and brochure groups [105,115]. Similarly, the Lung Screen Uptake Trial (LSUT) found no significant difference in completion rates attributable to an information film [16,109].

In some cases, the detailed information provided by DAs (specifically regarding false positives and low absolute benefit) can lead patients to opt out of screening [48,59]. Furthermore, personalized risk calculators that reduce a patient’s perceived risk may paradoxically fail to decrease screening interest due to “motivated reasoning” [116].

#### 5.2.4. Tailoring Tools to Smoking Status and Specific Barriers

Standardized materials often fail to address the specific psychosocial barriers of high-risk populations. Tailoring tools to a patient’s specific profile is a critical strategy to enhance relevance and effectiveness. “Lung Talk” utilizes algorithms to alter its script and message framing based on whether the user is a current or former smoker [40,113]. This is crucial because current smokers often hold different perceptions, such as higher fatalism or fear of judgment [73,108]. Tailored interventions focus on optimizing the behavioral response to risk (efficacy) rather than just elevating risk perception, which can induce avoidance [79].

“Lung Talk” also tailors content based on user-selected barriers; if a user selects “cost/insurance” or “worry,” the tool plays a specific video addressing that exact concern [40]. The “Patient Voices” video specifically addresses “tobacco-related stigma” to provide social support [62].

Despite these advances, tools like “Lung Talk” improved perceived benefits and self-efficacy but did not significantly reduce perceived barriers compared to non-tailored information in some pilots [113].

### 5.3. Targeted Outreach

#### 5.3.1. Culturally Sensitive Materials and “Citizen Scientists”

To effectively reach underserved communities and address deep-seated medical mistrust, outreach strategies must be tailored to the specific social and cultural values of the target population. In emerging qualitative and pilot initiatives, researchers are employing “citizen scientists”—lay community members trained in research methods—to assist in the “co-creation” of culturally targeted DAs, particularly for African American men [52]. This process utilizes specific targeting strategies, including “peripheral” (using images salient to the group), “evidential” (presenting group-specific cancer rates), “linguistic” (using community-specific language), and “sociocultural” (incorporating cultural beliefs and values) adaptations [52].

In a recent cluster randomized controlled trial protocol in Aotearoa, New Zealand, trials engaging Indigenous Māori populations employ “whānau engagement coordinators” to provide cultural support and a “warm handover,” ensuring a culturally safe environment [106]. Similarly, observational data from clinic programs shows outreach to Native American communities has included “bundles of traditional medicine (sage, cedar, or sweetgrass)” and storybooks about traditional tobacco to differentiate it from commercial use [82]. For Hispanic populations, qualitative research indicates a preference for educational materials delivered through “personal stories” rather than purely clinical data [55].

Expert consensus and narrative reviews suggest that collaboration with trusted entities, such as faith-based organizations and community clinics, is more effective than impersonal healthcare correspondence [29,31]. For example, the PROSPR-Lung consortium engaged communities by visiting churches and health fairs [35], and recruitment materials often feature “culturally relevant images of patients” to enhance relatability [47].

#### 5.3.2. CHWs

These individuals serve as vital links between the healthcare system and the community, providing culturally tailored education and navigating patients through barriers like transportation and scheduling [28,93].

In pilot studies, CHW-delivered outreach successfully increased LCS knowledge and decreased stigma [92]. Broader review data indicate they significantly improve cancer awareness and screening rates [31].

#### 5.3.3. Mobile Screening Units

Mobile LDCT platforms are emerging as a powerful solution to bridge the gap between healthcare systems and underserved communities, particularly in rural areas or regions with limited medical infrastructure. Mobile units are effective in reaching high-risk individuals who are uninsured, underinsured, or residing in rural “screening deserts” [27,53]. For rural Veterans facing transportation challenges, qualitative assessments reveal healthcare teams identified mobile screening (e.g., a “CAT scan on a truck”) as a critical facilitator [117].

Locating scanners in convenient sites, such as supermarket car parks (as seen in the UK’s “Lung Health Check” pilots) or shopping centers, successfully engages populations in the most deprived socioeconomic quintiles, according to data from pilot programs and randomized controlled trials [34,67]. Observational data indicate mobile programs in the U.S. have reached populations with higher proportions of racial and ethnic minorities compared to fixed hospital sites [27].

#### 5.3.4. Rebranding as a “Lung Health Check” (LHC)

To overcome the stigma (e.g., self-blame) and fear associated with a cancer diagnosis, programs are rebranding the screening invitation. Strategies include framing the intervention as a LHC or wellness service. This approach, used in the Yorkshire Lung Screening Trial, includes a broader respiratory assessment (e.g., spirometry) and avoids immediate reference to cancer to recruit higher-risk individuals who might otherwise avoid the appointment due to fatalism [67,118].

The LSUT utilized a “M.O.T. for your lungs” concept (referencing the UK’s annual vehicle suitability test) to normalize the offer as a routine check-up [58]. While this “low-burden” leaflet did not improve uptake overall, it was relatively more effective at engaging individuals in the most socioeconomically deprived areas [119]. Effective materials avoid “scare tactics” or blame, focusing instead on hope, family, and the benefits of early detection [42].

#### 5.3.5. Social Media and Digital Outreach

Digital platforms offer a method to raise awareness and identify eligible candidates outside of traditional clinical encounters. The protocol for the INSPIRE-Lung randomized controlled trial leverages Facebook targeted advertisements (FBTAs) to recruit eligible individuals by targeting users based on age (50+), location, and keywords related to smoking [40]. This “precision marketing” shifts the focus to before the individual enters the healthcare system [40].

Retrospective observational studies have shown that paid digital campaigns on Facebook, Google, LinkedIn, and Twitter have been associated with increased visits to institutional websites and increased weekly scheduled exams [92,120]. FBTAs have specifically been successful in recruiting a racially diverse national sample of older smokers, as demonstrated in a pilot randomized controlled trial [113].

#### 5.3.6. Accessibility and Health Literacy

Ensuring materials are accessible to those with lower health literacy is essential for equitable outreach. Analysis reveals that standard LCS materials are often too complex, requiring a college degree to understand, whereas they should be written at a 3rd to 7th-grade level [42]. Qualitative data from focus groups shows participants expressed a preference for simple language and pictograms, noting that standard materials often contained “words a little too big” [48].

To address this, programs utilize “low information burden” leaflets that provide just enough information to prompt attendance without overwhelming the user [67,119]. Randomized clinical trials have demonstrated that video-based DAs (e.g., “Lung Talk”, “Patient Voices”) with narration and animation are effective for populations with lower education levels, helping to improve preparedness and reduce tobacco-related stigma [62,115]. However, some low-income populations still prefer paper pamphlets over web-based tools due to the “digital divide” [48].

### 5.4. Communication Strategies and Therapeutic Alliance

#### 5.4.1. Therapeutic Alliance and Provider Trust

To foster the therapeutic alliance necessary for adherence, communication strategies should validate the patient as a whole person, avoiding ‘blaming’ language regarding smoking history [73,107]. Even when patients do not fully understand specific risks or the rationale for active surveillance, their faith in the provider’s expertise bridges these knowledge gaps [48,99].

A strong alliance acts as a buffer against anxiety; patients report satisfaction with their care despite limited knowledge because they trust their doctor would “call me back if it was serious” [68]. Conversely, a lack of alliance or the perception that a provider is not genuinely concerned can lead to patients discounting recommendations and “falling through the cracks” [68,107].

#### 5.4.2. Person-Centered Communication

Effective communication strategies must validate the patient as a whole person, addressing the unique emotional burden of LCS regarding stigma and anxiety. Clinicians are encouraged to adopt strategies that foster partnership, such as explicitly asking “What’s on your mind?” and validating distress as a common reaction [68]. Patients value “good bedside manner” where they are treated “as a human being” rather than “an object” or “an ATM machine” [48].

To avoid alienation, communication must avoid “scare tactics,” “blaming the victim,” or “beating up” on patients regarding their smoking habits [42,48,64]. Strategies include rebranding the process as a LHC to reduce stigma and focusing on the benefits of early detection [42,64]. Tools like the “mHealth TLC” virtual companion and “Patient Voices” videos are designed to manifest empathy, validate feelings of self-blame, and normalize the screening process [62,72].

There is a distinct tension between clinical efficiency and patient needs regarding results communication. While clinicians often view mailed letters as efficient for low-risk results, patients strongly prefer verbal conversations to feel “heard,” ask questions, and receive “handholding” through the evaluation plan [50,57].

Communication should be adapted to the patient’s style (e.g., “data people” vs. those preferring general recommendations) and health literacy levels [61]. Programs utilize “low burden” information materials that minimize cognitive load to engage patients without overwhelming them with statistics, before a supportive discussion can take place [16,58].

Effective recruitment often begins with low-burden methods (automated letters, texts, or portal messages) and escalates to more direct, resource-intensive contact for non-responders [62,119]. The Larch Study progresses from automated notifications to “automated voice-call reminders,” and finally to personal scheduling assistance by staff [62]. The mFOCUS trial moves from electronic health record reminders (Step 1) to population health outreach (Step 2) and finally to intensive patient navigation with personal calls (Step 3) [87]. The Yorkshire Lung Screening Trial uses a “staggered” schedule involving pre-invitation, formal invitation, and reminder letters [67]. Operational guidelines suggest formal attempts at 30, 60, and 90 days before notifying the primary care provider [13].

Evidence confirms that personal contact and reminders are far more effective than passive methods. One study found 59% screening completion among those receiving a phone call from a program manager versus only 9% for those receiving mailed materials alone [92]. Another study found 64% adherence in a reminder group compared to 0% in a group receiving no specific reminder [36]. Re-invitation letters alone have also been shown to significantly improve uptake, particularly among under-represented groups [121].

#### 5.4.3. Addressing Medical Mistrust

Despite clinician fears that using words like “cancer” causes anxiety, patients generally prefer transparency regarding risks (including overdiagnosis and false positives) and the rationale for surveillance [50,68]. Withholding information is often ineffectual and can erode trust, whereas clear explanations can reverse a patient’s initial decision to decline screening [49,68]. Failure to ensure appropriate follow-up is described as a violation of the trust patients place in health systems [87].

The use CHWs or navigators who share the same racial or social background as the patient can bridge the gap between the healthcare system and the community [31,92]. Acknowledging low trust in expert entities, some studies leverage social media (“precision marketing”) to meet patients in their own digital environments, bypassing initial skepticism of institutional cold-calls [40].

While patients may mistrust the healthcare system generally, they often hold “strong trust” in their specific local clinic or provider. Interventions that leverage this personal connection—where clinicians demonstrate a vested interest in the patient’s personal life—are key facilitators for groups like Urban Native Americans and Black Veterans [49,82].

### 5.5. Leveraging the Screening Process as a “Teachable Moment”

Participation in lung screening programs has been associated with quit rates superior to those observed in the general population. Similarly, the NELSON trial observed lower smoking rates in screened participants compared to the general population [23].

Programs are encouraged to integrate evidence-based interventions into the screening workflow. To capitalize on the motivation discussed in Section 3, programs should implement the ‘5 A’s’ model (Ask, Advise, Assess, Assist, Arrange) and integrate opt-out pharmacotherapy directly into the screening workflow. The “5 A’s” model is a standard framework recommended for every patient interaction to promote cessation [13,33]. The CMS mandates smoking cessation counseling for current smokers as a requirement for reimbursement [76,81].

To optimize cessation, pharmacotherapy such as nicotine replacement therapy (NRT) and varenicline should be offered to participants who are still smoking, as nicotine dependence is high in this cohort [32,33]. The “teachable moment” may be most potent when patients receive abnormal results. Studies indicate that positive screening results directly correlate with increased rates of smoking cessation, whereas negative results do not necessarily encourage continued smoking (the “license to smoke” phenomenon), though they may require specific communication strategies to maintain retention in cessation programs [13,81].

## 6. Limitations

This review has several limitations that should be acknowledged. Although a structured and predefined search strategy was employed, the review is narrative in nature and does not follow a formal systematic review protocol with risk-of-bias assessment or meta-analytic synthesis. As such, the findings are subject to potential selection bias and heterogeneity across included study designs, populations, and healthcare settings. The exclusion of non-English publications and conference abstracts may have limited the inclusion of emerging implementation data, particularly from low- and middle-income countries. Additionally, much of the available evidence originates from the United States and other high-income settings, which may affect the generalizability of findings to healthcare systems with different reimbursement models and infrastructural capacities. Finally, several conclusions rely on self-reported patient and provider perceptions, which may introduce recall or social desirability bias.

## 7. Priorities for Future Research

While much of the current literature focuses on initial uptake, the mortality benefit of LCS is predicated on consistent annual participation over decades. Future research must prioritize the study of long-term adherence beyond the baseline (T0) and first annual (T1) rounds to address the significant “drop-off” observed in clinical practice, where adherence can fall to as low as 2.8% by the second year [35,96]. Longitudinal studies are needed to identify specific behavioral and system-level determinants that sustain engagement over time, particularly for patients with negative baseline screens who are at the highest risk of attrition due to a false sense of security [38,65].

There is an urgent need to move beyond “one-size-fits-all” annual protocols toward personalized, adaptive screening. Research should investigate whether low-risk individuals (e.g., those with negative baseline scans) can safely transition to biennial screening intervals to reduce radiation exposure, patient anxiety, and program costs [24,32,80]. Concurrently, rigorous trials are required to determine the cost-effectiveness and clinical placement of blood biomarkers (liquid biopsy) and artificial intelligence for nodule classification. Specifically, studies must define whether these tools function best as “rule-in” or “rule-out” tests to minimize false positives and manage the “hot topic” of incidental findings [23,32,122].

Future research must address the structural inequities inherent in current eligibility guidelines. Studies are needed to validate the implementation of individualized risk prediction models (e.g., PLCOm2012) in primary care settings compared to fixed criteria (e.g., USPSTF), specifically to ensure the inclusion of high-risk African American populations who are often excluded by pack-year thresholds [52,84]. Additionally, given the rising incidence of lung cancer in non-smokers, research is needed to develop screening protocols for never-smokers, particularly Asian women and those with environmental exposures [29,32].

Finally, further investigation is required to resolve the debate regarding the behavioral impact of screening results. Studies should examine whether negative screening results induce a “license to smoke” in the long term and evaluate standardized protocols for integrating opt-out smoking cessation interventions directly into the radiology workflow to maximize the “teachable moment” [13,69,81]. Furthermore, future studies should investigate the effectiveness of embedding structured cessation frameworks, such as the “5 A’s” approach, and immediate access to pharmacotherapy (e.g., varenicline, NRT) directly within screening workflows, compared with referral-based or advisory models [13,33]. Addressing these questions is essential to identifying scalable approaches that maximize the preventive potential of lung cancer screening.

## 8. Conclusions

Suboptimal uptake and poor longitudinal adherence to LCS are driven by a complex interplay of patient- and provider-level factors that extend well beyond initial eligibility or access to imaging. Together, these factors contribute to substantial attrition after baseline screening, particularly among socioeconomically disadvantaged and historically underserved populations.

Addressing these challenges requires a shift from conceptualizing lung cancer screening as a one-time radiologic event toward a longitudinal, programmatic model of care that supports patients across repeated screening rounds. Within such a framework, patient navigation emerges as a critical facilitator, bridging patient and provider gaps by addressing logistical, psychosocial, and informational barriers, reinforcing trust, and ensuring continuity of care. Equally important is the systematic integration of smoking cessation interventions into screening workflows, leveraging the screening encounter as a teachable moment for primary prevention. Aligning patient navigation, tobacco dependence treatment, and longitudinal follow-up within structured screening programs is essential to improving adherence, reducing disparities, and ultimately realizing the full mortality benefit of lung cancer screening in real-world practice. While much of the available evidence derives from U.S. and European healthcare systems, careful consideration is required when extrapolating these findings to low- and middle-income settings, where differences in infrastructure, financing models, and sociocultural dynamics may substantially shape screening feasibility and adherence patterns.

## Figures and Tables

**Table 1:** Summary of mortality reduction in key LCS trials [14,22].

Trial Name	Number of Participants	Screening Interval/Duration	Follow-Up Period	Lung Cancer Mortality Reduction (Primary Outcome)	Refs
NLST (National Lung Screening Trial)	53,454	Annual (3 rounds)	Median 6.5 years	20% reduction (vs. chest X-ray)	[13,14,22]
NELSON Trial	15,822	Increasing intervals (0, 1, 2, and 2.5 years; 4 rounds)	10 years	24–26% reduction in men (vs. no screening); larger reduction observed in women (39–61%)	[14,22,23]
MILD (Multicentric Italian Lung Detection)	4099	Annual or Biennial (median 6 annual or 3 biennial screens)	10 years	39% reduction at 10 years (vs. no screening); no difference found between annual vs. biennial intervals	[22,23,24]
LUSI (German Lung Cancer Screening Intervention)	4052	Annual (5 rounds)	Median 8.8 years	No statistically significant reduction overall; significant reduction observed in women (HR 0.31)	[14,22,23]
DANTE (detection and screening of early lung cancer)	2472	Annual (5 rounds)	Median 8.4 years	No statistically significant reduction	[14,22]
DLCST (Danish Lung Cancer Screening Trial)	4104	Annual (5 rounds)	Median 9.5 years	No statistically significant reduction	[14,22]
ITALUNG (Italian Lung Cancer Screening)	3206	Annual (4 rounds)	Median 8.5 years	No statistically significant reduction (30% reduction observed but not statistically significant)	[14,22]
UKLS (UK Lung Cancer Screening Pilot)	4055	Single screen (1 round)	Median 10 years	Non-significant reduction in primary analysis due to pilot design/sample size (RR 0.86)	[14,22,25]

Note: While smaller European trials (DANTE, DLCST, ITALUNG) individually did not reach statistical significance regarding mortality reduction, largely due to sample size and power, meta-analyses of these trials combined generally support the efficacy of LDCT screening [14]. HR—hazard ratio; RR—relative risk.

**Table 2:** Thematic categorization of patient-reported barriers to LCS from qualitative studies [45,59].

Barrier Domain	Specific Themes	Examples
Individual/psychological	Knowledge avoidance and fear of disease	Anxiety regarding a potential diagnosis; Preference for ignorance over knowing bad news; Fear of treatment outcomes.
	False positive worry	Anxiety regarding the stress of potential misdiagnosis or inconclusive results.
	Fear of screening procedure	Claustrophobia or anxiety regarding the physical scan.
	Denial of risk	Belief that lack of symptoms equates to health.
System/practical	Cost & insurance misunderstanding	Real or perceived financial burden; Confusion regarding coverage; Inability to pay copays.
	Logistical barriers	Lack of time; Conflicts with work; Inconvenience of location.
	Confusion around results	Frustration with inconclusive findings or the “runaround” of diagnostic testing.
Cultural and beliefs	Fatalistic beliefs and perceived low value	Belief that screening makes no difference to the outcome; “What I don’t know won’t hurt me” attitude; Skepticism about benefits.
	Distrust	Suspicion of medical system motives (profit over care); Feeling marginalized or unheard by doctors.

**Table 4:** Changes in smoking behavior, attitudes, and cessation rates following LCS [13,14,24,77,78].

Domain	Metric/Population	Pre-Screening (T0)/Control Group	Post-Screening (T1)/Intervention Group	Notes	Ref.
Attitudes and motivation	Readiness to quit	32.9% (Ready in next 30 days)	N/A	25.7% of participants reported increased readiness to quit following screening (*p* < 0.001).	[77]
	Motivation to quit score (mean, scale 1–10)	6.5 (SD 2.3)	6.7 (SD 2.3)	Statistically significant increase in motivation (*p* < 0.05).	[77]
Consumption	Cigarettes per day (mean)	18.2 (SD 9.0)	16.7 (SD 9.1)	Statistically significant reduction in daily cigarette consumption (*p* < 0.001).	[77]
	Decreased smoking (study of 1060 adults)	N/A	45%	45% of smokers decreased smoking after the first screening; this was more typical in younger participants (<65 years).	[78]
Cessation rates (trial data)	Mayo Clinic screening program (longitudinal study)	5% to 7% (General population historical rate)	14% (Year 1) 22% (Year 2) 24% (Year 3)	Screening program participants exceeded general population quit rates; 98% of former smokers (>1 yr) remained tobacco-free.	[13]
	NELSON Trial (2-year follow-up)	N/A	16.6% (Screening arm) (control arm was 19.1%)	While rates were high, no significant difference was found between screening and control arms in this specific analysis.	[13,24]
	UKLS Trial (2-year follow-up)	21% (Control arm)	24% (Screening arm)	Net trial quit rate of 22% was significantly higher than the UK general population rate of 4%.	[24]
	UKLS Trial (2 weeks post-randomization)	N/A	RR 2.16 (vs. control)	Significantly higher quit rate in the LDCT screening group compared with control (95% CI 1.47 to 3.18).	[14]
Impact of test result	Abnormal/positive screen	N/A	41.9% (with 3 positive screens); 24.2% (with 1 positive screen)	Positive correlation between the number of positive results and smoking abstinence; suspicious findings were associated with higher cessation.	[13]
	Negative screen (false reassurance)	N/A	19.8% quit rate (with no positive exams)	Evidence argues against a “permission to smoke” phenomenon; negative screens did not lead to lower abstinence compared to general population rates.	[13,24]

Note: In [77] post-screening readiness was categorical, but significant intra-individual increases were observed.

**Table 5:** Patient characteristics associated with adherence to LCS protocols [28,38,56,65,94,95,96].

Predictor	Comparison	Association with Adherence/Nonadherence	Refs.
Smoking Status	Current vs. Former Smokers	Nonadherence: Current smokers were significantly more likely to be nonadherent compared to former smokers (RR 1.23; 95% CI 1.09–1.40).	[56,65,95]
Sex	Female vs. male	Mixed/Nonadherence: Meta-analysis found no significant difference (RR 0.99; 95% CI 0.85–1.15). However, individual studies have shown mixed results, with some finding males more adherent.	[56,96]
Race	White vs. non-white/Black	Adherence: White patients were twice as likely to adhere to screening compared to patients of other races (OR 2.0; 95% CI 1.6–2.6). Black patients demonstrated lower adherence to annual screening (aRR 0.73 in decentralized programs, without dedicated tracking staff like screening coordinators or navigators).	[38,94]
Education	College vs. no college/high school	Adherence: Completion of 4 or more years of college was associated with increased adherence (OR 1.5; 95% CI 1.1–2.1). Education > High School diploma associated with higher adherence (OR 1.87).	[65,94]
Screening result	Lung-RADS 3/4 (suspicious) vs. Lung-RADS 1/2 (negative)	Adherence: Patients with suspicious or abnormal findings were significantly more adherent to follow-up than those with negative screens (OR 3.8 for Lung-RADS 3; OR 14.0 for Lung-RADS 4).	[95]
Insurance type	Medicare/private vs. Medicaid/uninsured	Adherence: Patients with Medicare were more likely to adhere compared to Medicaid (OR 2.23) or dual-eligible patients. Uninsured patients or self-pay cohorts had higher nonadherence rates.	[56,65]
Program structure	Centralized vs. decentralized	Adherence: Centralized programs (with navigation/tracking) showed higher adherence (76.1% vs. 34.8%) and mitigated racial disparities compared to decentralized models.	[28,38]
Comorbidities	COPD diagnosis vs. No COPD	Adherence: Patients with a diagnosis of COPD were more likely to complete screening uptake/adherence (OR 1.13; 95% CI 1.06–1.20).	[96]

Note: COPD—chronic obstructive pulmonary disease; Lung-RADS—Lung Imaging Reporting and Data System; RR—Relative Risk; OR—Odds Ratio; aRR—adjusted Relative Risk.

**Table 6:** Disparities in LCS knowledge, comfort, and practice patterns between primary care providers and specialists (pulmonologists and oncologists).

Attitude/Practice Metric	Primary Care Providers/General Practitioners (PCP/GP)	Specialists (Pulm/Onc)	*p*-Value	Refs
Able to identify appropriate patients	63.8%	93.5%	<0.01	[100]
Feel comfortable counseling patients	51.4%	82.8%	0.01	[100]
Have sufficient time to counsel	14.3%	50.0%	<0.01	[100]
Confused about applying guidelines	63.8%	35.5%	0.01	[100]
Believe yearly screening is feasible	27.5%	86.7%	<0.01	[100]
Believe screening is NOT cost-effective	8.6%	29.0%	0.01	[100]
Aware that LDCT is an effective test	18.0%	81.0% (Onc)	<0.0001	[101]
Currently propose screening in practice	20.0%	53.0% (Pulm)	<0.001	[101]
Use inappropriate screening test (e.g., CXR)	93.0%	44.0% (Pulm)	<0.0001	[101]
Recommend correct annual screening interval	7.0%	76.0%	<0.0001	[101]
Propose screening for “all smokers” (Incorrect criteria)	55.0%	25.0%	0.04	[101]
Believe tobacco control MUST be associated with screening	52.0%	80–86%	<0.0001	[101]

## Data Availability

No new data were created or analyzed in this study. Data sharing is not applicable to this article.

## Supplementary Materials

The following supporting information can be downloaded at: https://www.mdpi.com/article/10.3390/diagnostics16071092/s1, Table S1: Population scale, screening intensity, and lung cancer incidence in major lung cancer screening trials: general population versus screening-eligible high-risk groups; Table S2: Illustrative patient sentiments/quotes relative to the barriers against their adherence to a lung cancer screening program with low-dose CT.

## Abbreviations

The following abbreviations are used in this manuscript:

CHW	Community health worker
CMS	Centers for Medicare & Medicaid Services
CXR	Chest X-ray
DA	Decision aid
DANTE	Detection And screening of early lung cancer
DLCST	Danish Lung Cancer Screening Trial
EHR	Electronic health record
FBTA	Facebook targeted advertisements
GP	General practitioner
HR	Hazard ratio
ITALUNG	Italian Lung Cancer Screening
LCS	Lung cancer screening
LDCT	Low-dose computed tomography
LHC	Lung Health Check
Lung-RADS	Lung Imaging Reporting and Data System
LSUT	Lung Screen Uptake Trial
LUSI	German Lung Cancer Screening Intervention
MILD	Multicentric Italian Lung Detection
NLST	National Lung Screening Trial
NRT	Nicotine replacement therapy
OR	Odds ratio
PCP	Primary care provider
RR	Relative risk
SDM	Shared decision-making
SDOH	Social determinants of health
SES	Socioeconomic status
UKLS	UK Lung Cancer Screening Pilot
USPSTF	U.S. Preventive Services Task Force
VHA	Veterans Health Administration
