# Supplementary material for: Patient and Healthcare Provider Barriers in the LDCT Lung Cancer Screening Continuum
Source: Diagnostics (Basel). 2026 Apr 4;16(7):1092. doi: 10.3390/diagnostics16071092 (PMC13073977; doi:10.3390/diagnostics16071092)
Supplement: Supplementary file 1 [file diagnostics-16-01092-s001.zip › diagnostics-4179475-supplementary.pdf]

diagnostics

MDPI

Review

Patient and Healthcare Provider Barriers in the LDCT Lung Cancer Screening Continuum

SUPPLEMENTARY MATERIALS

1. Supplementary Tables

Supplementary Table S1 Population scale, screening intensity, and lung cancer incidence in major lung cancer screening trials: general population versus screening-eligible high-risk groups.

Screening trial	Country	Year(s)	Country population	Eligible population (≥30 PY)	Participants per 100k (general)	Lung cancer incidence (general /100k)	Lung cancer incidence in eligible population (/100k)
NLST	United States	2002–2009	~305 M	~18.3 M (6%)	~17.5	~70	**~1,050**
NELSON	Netherlands & Belgium	2003–2015	~27.3 M	~1.6 M (6%)	~57.8	~60–65	**~950–1,050**
MILD	Italy	2005–2011	~59.0 M	~3.0 M (5%)	~6.9	~68	**~1,100**
LUSI	Germany	2007–2011	~82.0 M	~4.9 M (6%)	~4.9	~61	**~900–1,000**
DANTE	Italy	2001–2006	~58.5 M	~2.9 M (5%)	~4.8	~66	**~1,050**
DLCST	Denmark	2004–2010	~5.5 M	~0.44 M (8%)	~74.6	~82	**~1,200–1,300**
ITA-LUNG	Italy	2004–2006	~58.5 M	~2.9 M (5%)	~5.5	~66	**~1,050**
UKLS	United Kingdom	2011–2013	~63.0 M	~3.8 M (6%)	~6.4	~70	**~1,000–1,100**

*For NELSON, the ratio was calculated using the combined population of the Netherlands and Belgium during the recruitment period (~27.3 million).

Population data source: Population, surface area, and population density were derived from national statistical offices and Eurostat. Lung cancer incidence and annual case numbers were estimated from SEER (United States) and historical Eurostat/GLOBOCAN datasets, using mid-period values corresponding to each screening trial.

1. Population size, country area, and population density (temporal alignment)

Europe

Eurostat - Population on 1 January by age and sex. Eurostat Database, 2000–2015. https://ec.europa.eu/eurostat

Eurostat – GISCO. Geographical data: country surface area.

United States

United States Census Bureau. Intercensal Estimates of the Resident Population, 2000–2010. https://www.census.gov

2. Lung cancer incidence – general population

Europe and global

Ferlay, J., I. Soerjomataram, M. Ervik, et al. *GLOBOCAN 2012: Estimated Cancer Incidence, Mortality and Prevalence Worldwide in 2012 v1.0*. 2013. https://publications.iarc.who.int/Databases/Iarc-Cancerbases/GLOBOCAN-2012-Estimated-Cancer-Incidence-Mortality-And-Prevalence-Worldwide-In-2012-V1.0-2012.

Ferlay, J., E. Steliarova-Foucher, J. Lortet-Tieulent, et al. "Cancer Incidence and Mortality Patterns in Europe: Estimates for 40 Countries in 2012." *European Journal of Cancer* 49, no. 6 (2013): 1374–403. https://doi.org/10.1016/j.ejca.2012.12.027.

Ferlay, Jacques, Hai-Rim Shin, Freddie Bray, David Forman, Colin Mathers, and Donald Maxwell Parkin. "Estimates of Worldwide Burden of Cancer in 2008: GLOBOCAN 2008." *International Journal of Cancer* 127, no. 12 (2010): 2893–917. https://doi.org/10.1002/ijc.25516.

United States

Howlader N, Noone AM, Krapcho M, et al. *SEER Cancer Statistics Review, 1975-2012*. National Cancer Institute, 2014. http://seer.cancer.gov/archive/csr/1975_2012/.

3. Size of the Screening-Eligible Population (≥30 Pack-Years)

United States

The National Lung Screening Trial Research Team. "Reduced Lung-Cancer Mortality with Low-Dose Computed Tomographic Screening." *New England Journal of Medicine* 365, no. 5 (2011): 395–409. https://doi.org/10.1056/NEJMoa1102873.

These publications document that the NLST-eligible population represents approximately 6–7% of the general population.

Europe

De Koning, Harry J., Carlijn M. Van Der Aalst, Pim A. De Jong, et al. "Reduced Lung-Cancer Mortality with Volume CT Screening in a Randomized Trial." *New England Journal of Medicine* 382, no. 6 (2020): 503–13. https://doi.org/10.1056/NEJMoa1911793.

Oudkerk, Matthijs, Anand Devaraj, Rozemarijn Vliegenthart, et al. "European Position Statement on Lung Cancer Screening." *The Lancet Oncology* 18, no. 12 (2017): e754–66. https://doi.org/10.1016/S1470-2045(17)30861-6.

These sources provide robust estimates indicating that 5–8% of Western European populations meet heavy-smoking eligibility criteria.

4. Lung Cancer Incidence in the Screening-Eligible Population

Bach, Peter B., Joshua N. Mirkin, Thomas K. Oliver, et al. "Benefits and Harms of CT Screening for Lung Cancer: A Systematic Review." *JAMA* 307, no. 22 (2012): 2418. https://doi.org/10.1001/jama.2012.5521.

Gage, J. C., M. Schiffman, H. A. Katki, et al. "Reassurance Against Future Risk of Precancer and Cancer Conferred by a Negative Human Papillomavirus Test." *JNCI Journal of the National Cancer Institute* 106, no. 8 (2014): dju153–dju153. https://doi.org/10.1093/jnci/dju153.

Tammemägi, Martin C., Hormuzd A. Katki, William G. Hocking, et al. "Selection Criteria for Lung-Cancer Screening." *New England Journal of Medicine* 368, no. 8 (2013): 728–36. https://doi.org/10.1056/NEJMoa1211776.

These studies consistently report lung cancer incidence rates of approximately 900–1,300 cases per 100,000 eligible individuals per year.

5. Trial-Specific European References

Becker, Nikolaus, Erna Motsch, Anke Trotter, et al. "Lung Cancer Mortality Reduction by LDCT Screening—Results from the Randomized German LUSI Trial." *International Journal of Cancer* 146, no. 6 (2020): 1503–13. https://doi.org/10.1002/ijc.32486.

Field, John K., Daniel Vulkan, Michael P. A. Davies, et al. "Lung Cancer Mortality Reduction by LDCT Screening: UKLS Randomised Trial Results and International Meta-Analysis." *The Lancet Regional Health – Europe* 10 (November 2021). https://doi.org/10.1016/j.lanepe.2021.100179.

Hunger, Theresa, Eva Wanka-Pail, Gunnar Brix, and Jürgen Griebel. "Lung Cancer Screening with Low-Dose CT in Smokers: A Systematic Review and Meta-Analysis." *Diagnostics* 11, no. 6 (2021): 1040. https://doi.org/10.3390/diagnostics11061040.

Infante, Maurizio, Silvio Cavuto, Fabio Romano Lutman, et al. "A Randomized Study of Lung Cancer Screening with Spiral Computed Tomography." *American Journal of Respiratory and Critical Care Medicine* 180, no. 5 (2009): 445–53. https://doi.org/10.1164/rccm.200901-0076OC.

Pedersen, Jesper H., Haseem Ashraf, Asger Dirksen, et al. "The Danish Randomized Lung Cancer CT Screening Trial—Overall Design and Results of the Prevalence Round." *Journal of Thoracic Oncology* 4, no. 5 (2009): 608–14. https://doi.org/10.1097/JTO.0b013e3181a0d98f.

Methodological Notes

Screening trials target **high-risk subpopulations**, not the general population. Therefore, *participants per 100,000 inhabitants* and *lung cancer incidence per 100,000 inhabitants*

are not directly comparable metrics, but together contextualize trial scale vs. disease burden.

Ratio calculation:

$$\text{Participants per 100,000 inhabitants} = \frac{\text{Number of Participants}}{\text{Country Population}} \times 100{,}000$$

These ratios do not reflect screening coverage or eligibility penetration, as trials targeted high-risk subpopulations (age, smoking history) rather than the general population. They are provided strictly for macro-level comparability of trial scale.

Density: Population density is calculated as:

$$\text{Density} = \frac{\text{Population}}{\text{Area}}$$

where, Area is the country area i.e. standard geopolitical surface (km²).

Lung cancer burden

Number of lung cancer cases/year is derived from SEER (USA) and Eurostat / GLOBOCAN historical estimates, averaged over the screening period. **Incidence rates** expressed per 100,000 inhabitants, age-standardized where possible in original sources, but here used as crude approximations for cross-country scale comparison.

Supplementary Table S2: Illustrative patient sentiments/quotes relative to the barriers against their adherence to a lung cancer screening program with low-dose CT.

Barrier domain	Illustrative patient sentiments/quotes	Reference
Individual / psychological	• "If I have lung cancer…basically I just don't want to know about it."	[1]
	• "It's part of the fear of having it done and then finding out that you do have it [lung cancer]... 'Oh, my God, I have it. I'm going to die.'"	[2]
	• "I think it's fear of the unknown – if I know, well then there's a scary response. You know you have to follow through and do more and more."	[1]
	• "I did schedule one and then after I read the print out... I canceled it…the false positives were so high... I thought that would be so stressful to think that you had it, and really you didn't."	[1]
	• "...that little tube was too much enclosure….And I got claustrophobia."	[2]

System / practical	• "I actually scheduled [a screening] and then they called me back and told me I needed to bring $300 with me. And I just didn't have it..."	[2]
	• "Once we got to the point where I realized it wasn't going to be covered by my insurance, that was basically the end of it…if it had been less expensive, I would have done it."	[1]
	• "I was still working at that time and I really didn't have time to get over there during the week and so I haven't had it done."	[1]
	• "...you have just put me through all this mind bending and tests and running all over the place... Tell me once and for all, do I have something or do I not?"	[2]
Cultural / beliefs	• "I feel like if it's my time, I don't want to know about it... I'm like what I don't know won't hurt me."	[2]
	• "It could show me if I had lung cancer and – what are they going to do?...screening for it doesn't really make any difference because I'll either come down with lung cancer or I won't."	[1]
	• "I'm kind of like the person that thinks doctors and insurance companies are in cahoots anyway... It's like they want to spend less money on you as possible."	[2]
	• "What is it going to do? What is it going to prove? That I don't have it right now. But in five years I could end up developing lung cancer from my past exposure."	[1]

References

1. Carter-Harris, L.; Brandzel, S.; Wernli, K.J.; Roth, J.A.; Buist, D.S.M. A Qualitative Study Exploring Why Individuals Opt out of Lung Cancer Screening. *FAMPRJ* **2017**, cmw146, doi:10.1093/fampra/cmw146.
2. Gressard, L.; DeGroff, A.S.; Richards, T.B.; Melillo, S.; Kish-Doto, J.; Heminger, C.L.; Rohan, E.A.; Allen, K.G. A Qualitative Analysis of Smokers' Perceptions about Lung Cancer Screening. *BMC Public Health* **2017**, *17*, 589, doi:10.1186/s12889-017-4496-0.

Disclaimer/Publisher's Note: The statements, opinions and data contained in all publications are solely those of the individual author(s) and contributor(s) and not of MDPI and/or the editor(s). MDPI and/or the editor(s) disclaim responsibility for any injury to people or property resulting from any ideas, methods, instructions or products referred to in the content.
